# Microfluidic dissociation of spleen to recover immune cells for biotechnology and single cell analysis

**DOI:** 10.3389/fimmu.2026.1819465

**Published:** 2026-07-29

**Authors:** Jeremy A. Lombardo, David Zalazar, Marzieh Aliaghaei, Jered B. Haun

**Affiliations:** 1 Department of Biomedical Engineering, University of California, Irvine, Irvine, CA, United States; 2 Department of Chemical and Biomolecular Engineering, University of California, Irvine, Irvine, CA, United States; 3 Department of Materials Science and Engineering, University of California, Irvine, Irvine, CA, United States; 4 Chao Family Comprehensive Cancer Center, University of California, Irvine, Irvine, CA, United States; 5 Center for Advanced Design and Manufacturing of Integrated Microfluidics, University of California, Irvine, Irvine, CA, United States

**Keywords:** dissociation, microfluidic, single cell, spleen, splenocyte

## Abstract

**Introduction:**

Splenocytes are important for various applications in medicine and biotechnology, but isolation methods are often limited by low yield, poor viability, bias towards certain cell subtypes, and degradation of surface marker expression. Here, we employ a microfluidic device to dissociate spleen tissue and isolate single splenocytes using non-enzymatic, enzymatic, and combined approaches.

**Methods:**

Spleen samples were minced and digested on-chip using enzyme-free buffer to assess short-term release of predominantly non-adherent CD45+ leukocyte populations. We then evaluated isolation of dendritic cells and macrophages by comparing non-enzymatic and enzymatic digestion protocols over time. A combined protocol, consisting of a buffer washout followed by collagenase digestion, was then used to balance enzyme exposure and maximize recovery.

**Results:**

Microfluidic digestion yielded greater cell recovery and viability across non-adherent splenocytes (B cells and T cell subpopulations) and adherent splenocytes (dendritic cells, macrophages, and neutrophils) within the CD45+ leukocyte population, compared to conventional strainer grinding and collagenase digestion controls. Functional validation was performed for B cells, which were isolated using the final protocol and cultured for six days, demonstrating comparable proliferation, viability, and antibody production levels relative to controls.

**Discussion:**

Future studies will evaluate additional splenocyte subpopulations using functional assays and downstream applications in medicine and biotechnology.

## Introduction

**Figure 1:**
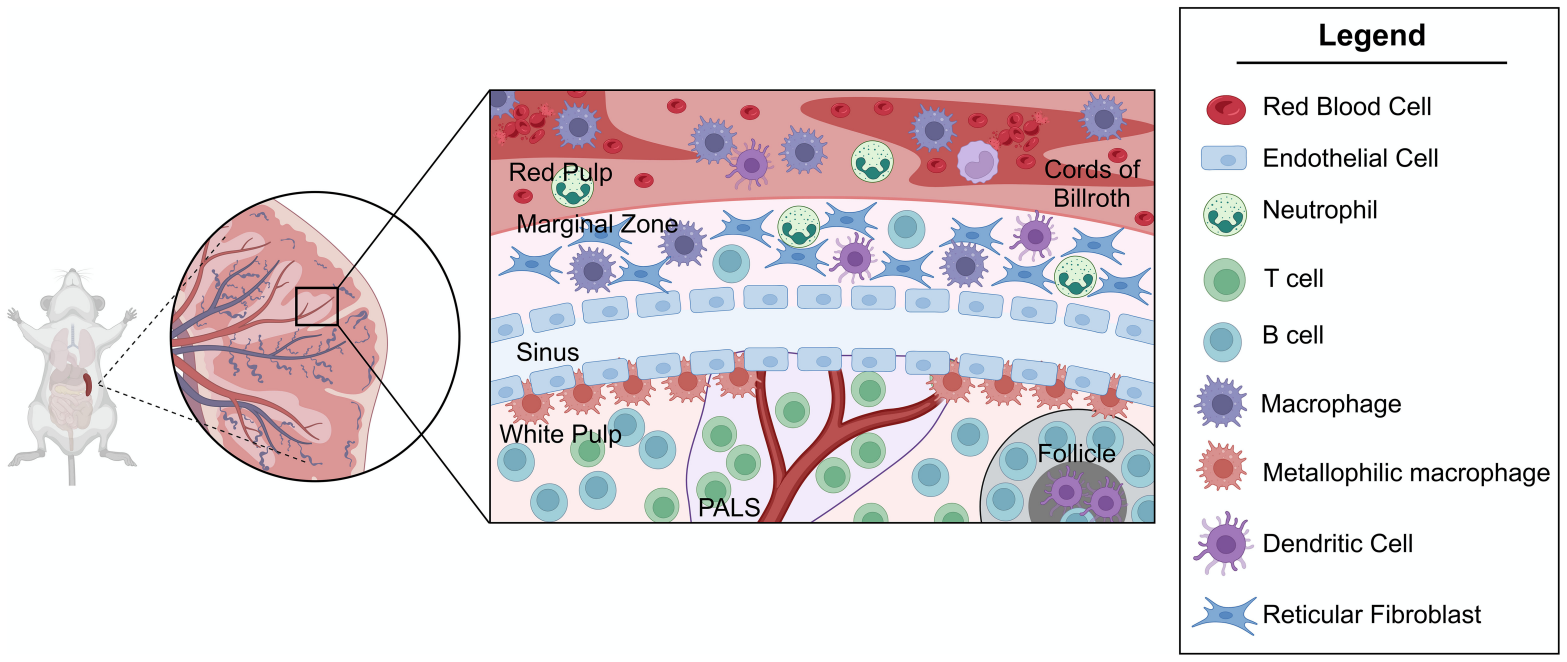
Schematic of the spleen. The spleen is comprised of two main compartments, the red pulp and the white pulp, each with distinct substructures. The red pulp primarily houses a collection of erythrocytes and macrophages, with interspersed cords of billroth that function as a filtration network to remove aged and damaged erythrocytes, as well as house abundant macrophages. Adjacent to the red pulp is the marginal zone, which contains B cells, macrophages, and other circulating immune cells organized within a structural network of reticular fibroblasts. The marginal sinus separates the white pulp from the marginal zone and is lined by specialized endothelial cells and metallophilic macrophages on the white pulp side. The white pulp is primarily composed of lymphocytes, with a T cell–rich periarteriolar lymphoid sheath (PALS) and B cell–rich follicles also containing follicular dendritic cells. Created in BioRender. Haun, J. (2026) https://BioRender.com/chztv8u.

The spleen is a critical organ for the immune system, as it includes both innate and adaptive responses (1). The unique structure and organization of the spleen also enables active filtration mechanisms and maintains erythrocyte homeostasis (2). The two main structures of the spleen, the red and white pulp, are composed of different cell types that carry out specific tasks (**Figure 1**). The red pulp houses an abundance of macrophages that are responsible for removing microorganisms, cellular debris, and aged erythrocytes. Red pulp also contains connective tissue that serves as in important reservoir for large populations of monocytes and neutrophils, many of which reside in an immature state for rapid deployment to sites of injury or infection where they play an essential role in the innate immune response (3, 4). The white pulp, meanwhile, is a lymphoid tissue composed of distinct regions of B and T cells that are surrounded by the marginal zone, which contains subsets of macrophages, dendritic cells, and more B cells. These white pulp cells are primarily responsible for initiating adaptive immune responses (1, 5).

Splenocytes are fundamentally important to many biotechnological applications. B cells have long been harnessed for production of monoclonal antibodies for use as reagents in various bioassays, and increasingly molecular drugs for treating cancer and other diseases (6–8). Moreover, splenocytes are actively being explored for use in cytotoxicity assays (9, 10), vaccine testing (11, 12), and adoptive cell transfer (13, 14). There is also rising interest in understanding the functional heterogeneity of immune cell subtypes within the spleen, which is increasingly being interrogated at single cell resolution (15–17). To isolate or analyze splenocyte subtypes, however, the tissue must first be dissociated into a suspension of single cells. Spleen dissociation methods are numerous and varied, but the most common methods are grinding on a strainer, shearing between microscope slides, or digesting with a proteolytic enzyme such as a collagenase or an enzyme cocktail such as Liberase. The method employed may simply be a matter of personal preference or may offer the best yield for a specific cell subtype of interest (18–20). Factors that influence isolation efficiency and kinetics include anatomical location within the spleen, as well as the strength of adherence to extracellular matrix proteins and/or other cell types. T cells, B cells, neutrophils, and some dendritic cell populations are considered to be nonadherent. Other dendritic cell populations, however, are weakly adherent, while macrophages are known to be strongly adherent (21). These classifications are primarily based on *in vitro* observations from culturing cells on plastic, but have also be informed by observations *in vivo* (20, 22). Given disparities in anatomical location and adhesion strength, it is generally expected that enzymatic digestion will liberate more cells across a broader scope of subtypes than purely mechanical approaches like grinding or shearing (18, 23). However, proteolytic enzymes can degrade surface markers and alter function of immune cells (24, 25). Most spleen dissociation methods are also entirely manual, which may impact inter-operator reliability, particularly for inexperienced users.

Preserving cellular numbers and states during dissociation is critical to ensure that isolated cells reflect conditions of the native *in vivo* conditions. This is especially important for the spleen, as dissociated cells are often used to characterize the immune landscape using methods such as single cell RNA sequencing. Dissociation artifacts can arise from disproportionate recovery of certain cell subtypes relative to others, either due to differential isolation or survival efficiencies, or alteration of gene expression, often related to stress-response programs. These artifacts can bias single cell RNA sequencing results (26, 27). Dissociation conditions may also influence the recovery of fragile immune subsets and compromise surface marker integrity, with important implications for studies focused on antigen presentation and immune phenotyping. Additionally, recent work has shown that tissue dissociation can generate macrophage-derived cellular remnants that remain associated with other cells, complicating phenotypic and molecular analyses (28, 29). Together, these findings suggest that tissue processing itself can shape downstream immune readouts and, if not carefully considered, may lead to inaccurate conclusions about splenic immune heterogeneity and cell state.

**Figure 2:**
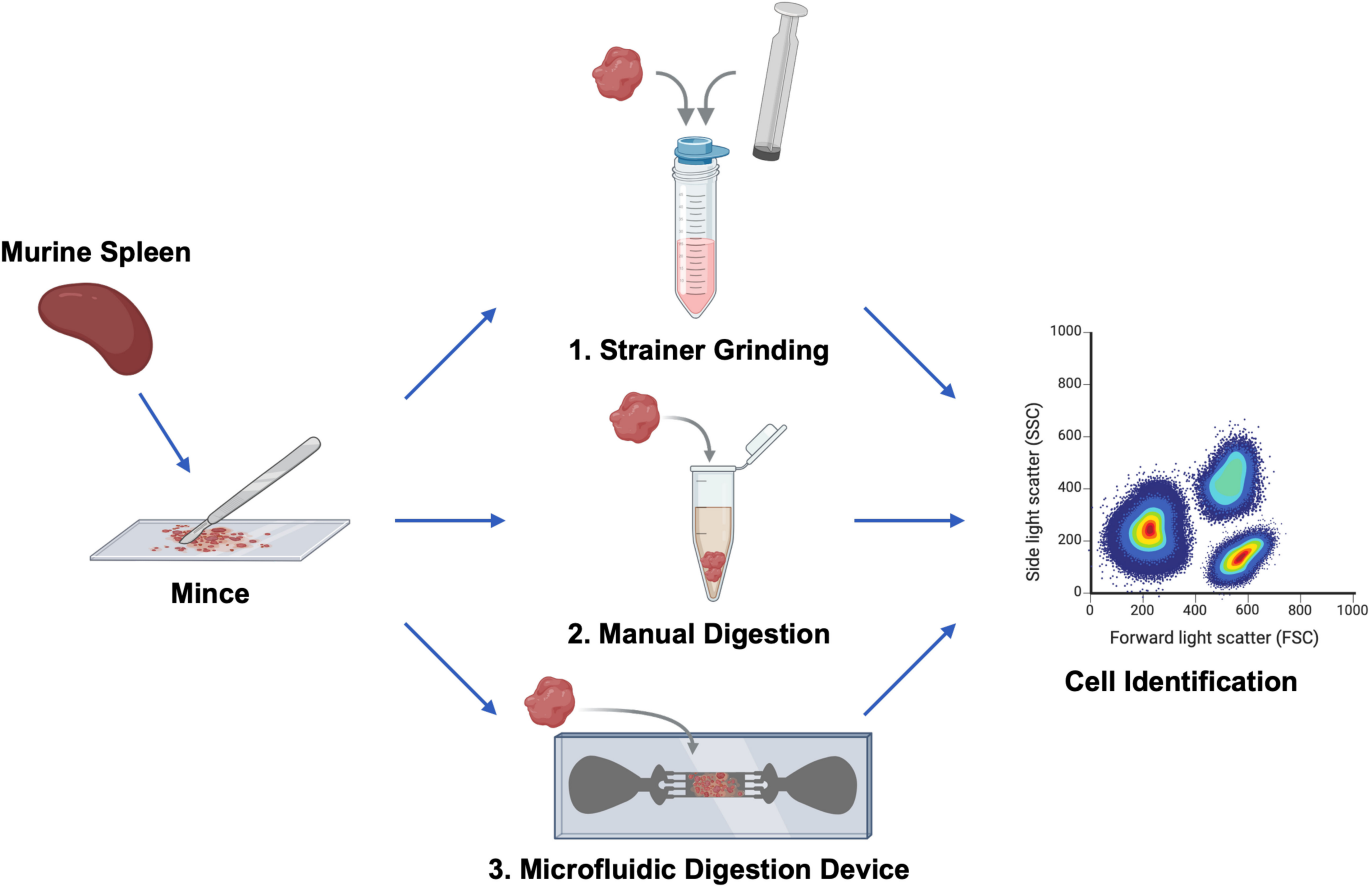
Experimental workflow for murine spleen dissociation and immune cell analysis. Harvested murine spleen was minced into ~1 mm³ pieces and processed by one of three methods: mechanical grinding through a mesh strainer, enzymatic digestion in a tube with collagenase type I for 60 min at 37 °C, or processing through a microfluidic digestion device. Following dissociation procedures, cells were analyzed by flow cytometry for immune subtype identification. Created in BioRender. Haun, J. (2026) https://BioRender.com/kt357ke.

Here, we employ a microfluidic device to dissociate murine spleen tissue and isolate single splenocytes. The digestion device was developed in previous works (30, 31), and has been shown to successfully isolate single cells faster and in higher numbers from murine kidney, mammary tumor, liver, and heart tissues utilizing both hydrodynamic shear forces and enzymatic digestion. Upon comparison to traditional spleen dissociation methods such as strainer grinding or manual digestion (**Figure 2**), we demonstrated that the device yields two- to three-fold more CD45+ leukocytes. Remarkably, this result could be achieved within a few minutes using both enzymatic digestion and a fully non-enzymatic protocol. We then establish a combined protocol, with a non-enzymatic period followed by digestion, which resulted in two to three-fold higher recovery for dendritic cells, macrophages, B cells, and T cells. Lastly, we validate B cell viability and function in terms of proliferation and antibody production. Based on these findings, our microfluidic device can be used to isolate splenocytes rapidly under non-enzymatic conditions and/or in substantially higher overall numbers using digestion. This work will advance biotechnology applications such as monoclonal antibody generation, cytotoxicity assays, vaccine testing, and adoptive cell transfer, as well as promote single cell analysis studies for mapping splenic cell subtype numbers and functional states.

## Results and discussion

### Characterizing non-enzymatic dissociation

We initially characterized cell recovery from the microfluidic device using non-enzymatic conditions. Due to the anatomy of the spleen and presence of many non-adherent or weakly adherent cell types, we hypothesized that hydrodynamic shear forces could be sufficient to release significant cell numbers, similar to standard grinding of spleen tissue against a strainer or between two glass slides. For these studies, freshly dissected murine spleen was minced using a scalpel into ~1 mm^3^ pieces and loaded into the device through the luer port. PBS buffer containing 1% BSA (PBS+) was then recirculated through the device using a peristaltic pump. The grinding method was used as a control, utilizing a 100 μm pore cell strainer and the plunger from a syringe.

**Figure 3:**
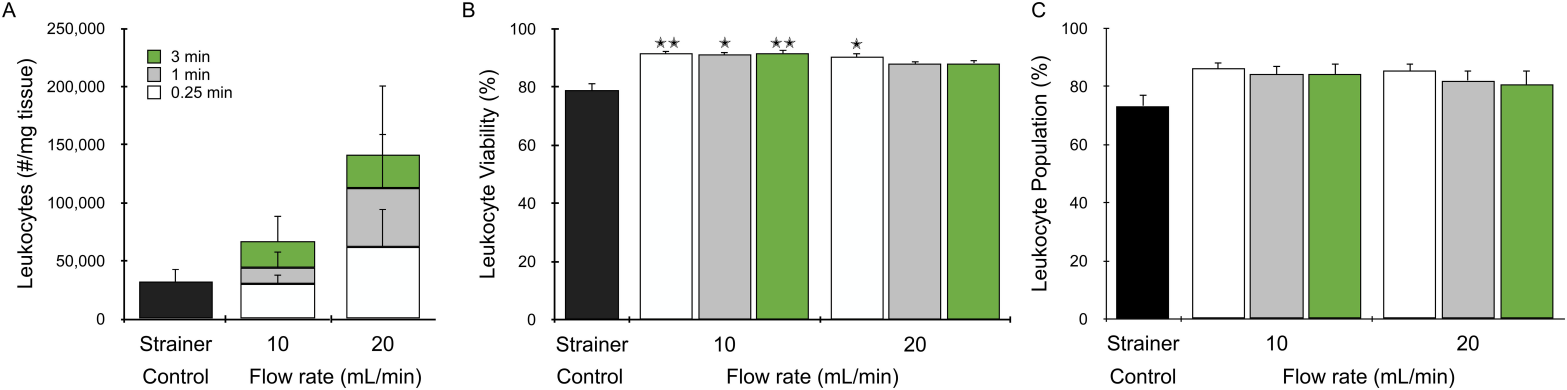
Non-enzymatic device operation. Minced spleen was loaded into the digestion device and PBS+ buffer was recirculated through at 10 or 20 mL/min flow rate. Results were compared to strainer control method. **(A)** Total leukocytes recovered, which was 4-fold higher for the drive at 20 mL/min relative to control. **(B)** Leukocyte viability was ~90% for all device conditions, while the control was ~80%. Mean tissue input: Strainer, 12.1 ± 3.2 mg; 10 mL/sec, 18.2 ± 2.7 mg; 20 mL/sec, 18.0 ± 2.7 mg. **(C)** Leukocyte population percentage was higher for 1-min and all device conditions compared to strainer. Error bars represent standard error of the mean from n = 3 independent experiments. * indicates p < 0.05 and ** indicated p <0.01 relative to strainer control.

We first explored using flow rates of 10 or 20 mL/min, which are comparable to previous works using our microfluidic devices (31–33). Samples were processed using an interval operation scheme, in which devices were run for a set time period, after which cell suspensions were flushed from the device using an equal volume of fresh buffer, and device operation was resumed. Interval times of 0.25, 1, and 3 minute were chosen to evaluate short term release expected for a non-enzymatic protocol. Cell suspensions were analyzed using flow cytometry to exclude red blood cells (RBCs), enumerate leukocytes broadly based on CD45 expression, and evaluate viability. After only 15 seconds of recirculation at 10 mL/min flow rate, >30,000 leukocytes were recovered per mg tissue, similar to the total number of leukocytes dissociated using the strainer control (**Figure 3A**). The second and third intervals recovered more leukocytes, for a net difference that was 2-fold more than the control. Increasing flow rate to 20 mL/min flow rate increased yield to >60,000 leukocytes/mg after 15 seconds, doubling the number dissociated using the strainer control and equaling the total number collected at 10 mL/min after 3 min. We found that >140,000 leukocytes/mg were obtained at 20 mL/min after 3 min, which was >4-fold more than the strainer control. Leukocyte viability was higher for all device conditions, and the overall difference was statistically significant between processing conditions (ART ANOVA, F_6,14_ = 6.25, p = 0.0023). Specifically, viability for the strainer control was less than 80%, while device-processed samples retained ~90% viability using both flow rates (**Figure 3B**). Since the 20 mL/min flow rate produced more leukocytes without compromising cell viability, this was chosen for all remaining studies.

We also confirmed that interval recovery preserved more cells than a 3 minute static operation mode (see **Supplementary Information**, **Supplementary Figure 1A**), although differences were not significant. Leukocyte viability was again higher for device conditions, and differed significantly across intervals (ART ANOVA, F_4,10_ = 10.79, p = 0.0012). Both 1-minute (Dunnett’s, p = 0.0004) and 2-minute (Dunnett’s test, p = 0.0043) intervals demonstrated statistically significant increases in viability compared to the strainer control (**Supplementary Figure 1B**). The benefit of interval operation mode has been shown in previous work during longer term tissue digestion in the microfluidic device, which was linked to damage caused by continuous recirculation (31). Taken together, these results demonstrate that non-enzymatic processing with the microfluidic device improves spleen dissociation and single leukocyte isolation relative to the standard strainer control method.

### Characterizing enzymatic dissociation

Next, we combined enzymatic digestion with device processing to liberate the maximum number of cells from spleen tissue. Results were compared to a standard digestion control, which consisted of mincing, incubating with collagenase type I for 60 min at 37 °C, and mechanically disaggregating by repeated pipetting and vortexing. For device conditions, collagenase type I was used as the recirculating fluid, flow rate was 20 mL/min, and intervals were 5, 15, and 60 min, similar to our previous work (31). Non-enzymatic conditions using PBS+ buffer was also included for comparison at matching time intervals. The flow cytometry panel was expanded to include surface markers for dendritic cells, as well as macrophages (**Tables 1**, **2**). Phenotypically, murine dendritic cells are defined as CD11c^hi^ and MHC class II+ (20, 34, 35), while macrophages are F4/80^hi^ (36). We chose to focus on these tissue-resident cell types as they can be more difficult to dissociate using purely mechanical methods, and thus often require enzymatic digestion for efficient dissociation (18–20). Collagenase type I was chosen based on our previous work with other tissues (30, 31). It is also on the harsher side, so as to release as many tissue-resident cells as possible, but we did not observe a change in MHC class II expression or primary panel surface marker integrity relative to the non-enzymatic strainer method (see **Supplementary Information**, **Supplementary Figure 2**).

**Table 1:** Primary flow cytometry panel.

Marker	Fluorophore	Clone
CD45	BV510	30-F11
CD11c	FITC	N418
F4/80	PE	BM8
TER119	AF647	TER-119
MHC class II	APC/Cy7	M5/114.15.2
Viability	7-AAD	N/A

**Table 2:** Cell types identified using primary flow cytometry panel.

Cell type	Surface markers
Leukocytes	CD45+, TER119-
Macrophages	CD45+, F4/80^hi^, TER119-
Dendritic cells	CD45+, CD11c^hi^, MHC class II+, TER119-

**Figure 4:**
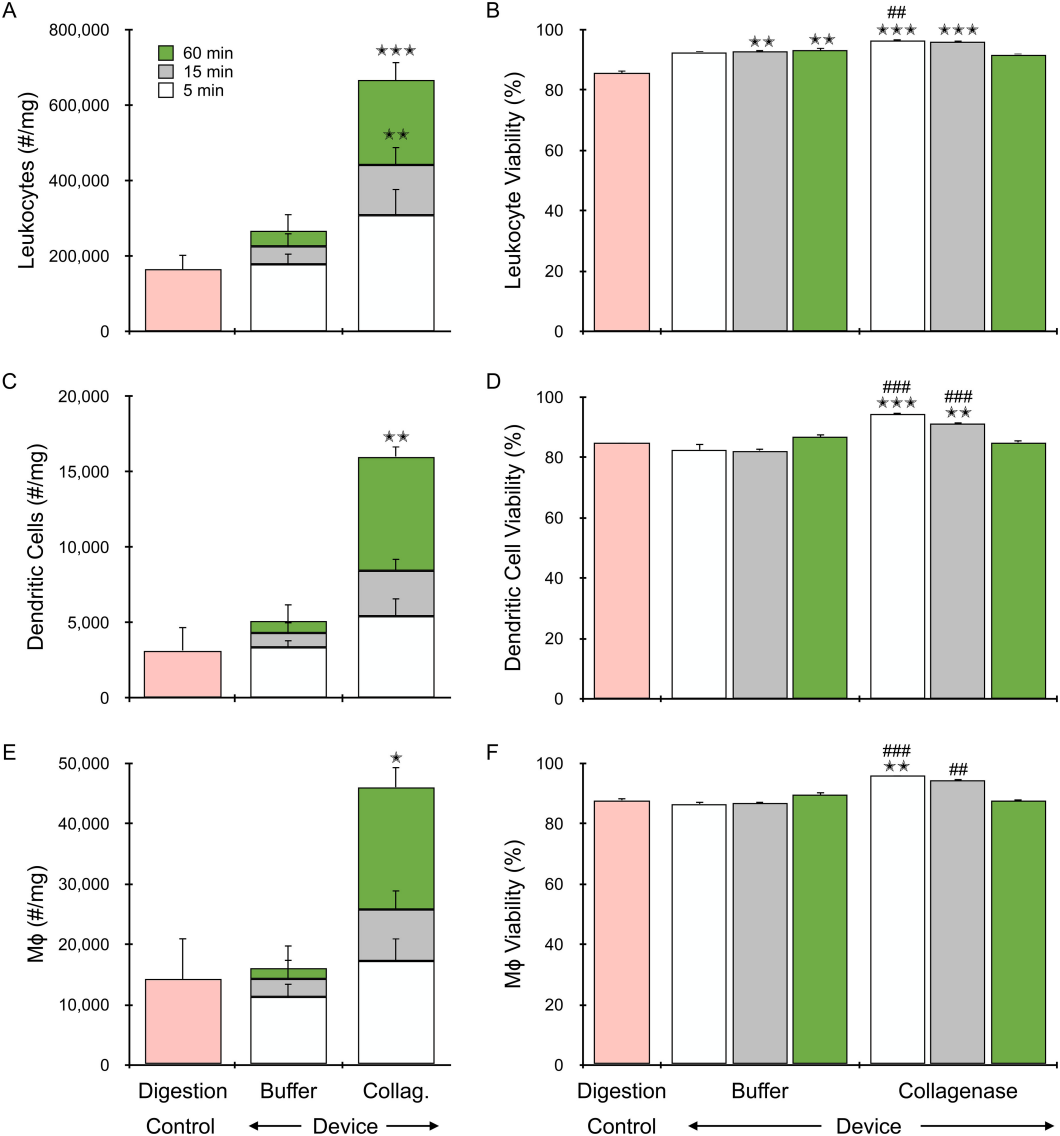
Enzymatic and non-enzymatic device operation. Minced spleen was processed in the digestion device using buffer or collagenase as the recirculating fluid. Results were compared to minced tissue that was digested with collagenase using a manual control method. Results are presented as **(A, C, E)** cell recovery and **(B, D, F)** viability for **(A, B)** total leukocytes, **(C, D)** dendritic cells, and **(E, F)** macrophages. Using buffer with the device produced similar cell yields to the control, while using collagenase increased recovery by 3-fold or more. Viability for all device conditions were comparable or greater than control. Error bars represent standard error of the mean from n = 3 independent experiments. Mean tissue input: Digestion, 23.4 ± 3.0 mg; Buffer, 23.4 ± 0.4 mg; Collagenase, 19.7 ± 0.5 mg. * indicates p < 0.05, ** indicates p < 0.01, and *** indicates p < 0.001 relative to collagenase control. ## indicates p < 0.01 and ### indicates p < 0.001 relative to time-matched buffer interval condition.

The manual digestion control generated ~160,000 leukocytes/mg (**Figure 4A**). Using buffer, the device intervals summed to ~270,000 leukocytes/mg, or >1.5-fold more than the control. A statistically significant effect was found based on processing method (ART ANOVA F_6,14_ = 7.76, p = 0.0008), suggesting that our microfluidic device can liberate at least as many immune cells using purely mechanical forces. Most leukocytes were freed within the first 5 min of processing, which equaled the number of cells recovered from the control after 60 min. Only modest numbers of additional leukocytes were recovered from the 15 and 60 min buffer intervals, as non-enzymatic processing efficacy quickly diminished. Using collagenase within the device, the intervals summed to >650,000 leukocytes/mg, or >4-fold more than the control, and this difference was statistically significant (Šidák’s test, p = 0.0009). Most leukocytes were again released during the initial 5 min interval, at ~300,000 leukocytes/mg, which was ~2-fold more than the control and comparable to the total recovery using the device with buffer. The 15 min interval resulted in >2.5-fold more than the control at ~440,000 leukocytes/mg (Šidák’s test, p = 0.0074). Taken together, these results demonstrate that the combination of collagenase and hydrodynamic forces within the device dramatically enhanced liberation of leukocytes. Viability was ~85% for the digestion control (**Figure 4B**), but remained >90% for the digestion device using both buffer and collagenase (ART ANOVA, F_6,14_ = 16.16, p < 0.0001).

Dendritic cells account for a small fraction of total leukocytes recovered, and as such the collagenase control produced only ~3,000/mg (**Figure 4C**). Despite these small cell numbers, results for the microfluidic device intervals using buffer and collagenase mirrored those observed for total leukocytes and exhibited significant differences across conditions (ART ANOVA, F_6,14_ = 5.15, p < 0.0055). Total recovery using collagenase was ~16,000 dendritic cells/mg, which was >5-fold more than the control (Šidák’s test, p = 0.0061) and >3-fold more than using buffer. Viability remained between 80 and 95% for all conditions (**Figure 4D**, ART ANOVA, F_6,14_ = 15.29, p < 0.0001). The 5 min collagenase interval had the highest viability at ~95% (Šidák’s test, p = 0.0008), which decreased moderately with additional processing time, back down to ~85% after 60 min. For macrophages, recovery differed significantly across processing conditions (**Figure 4E**). Both the 60 min collagenase control and total recovery using the digestion device with buffer produced ~15,000 cells/mg tissue (**Figure 4E**). Only 5 min was needed to match this value using collagenase with the device. Moreover, total recovery for device with collagenase was >3-fold higher than both control and device with buffer, however, only the control comparison was significant (Šidák’s test, p = 0.0227). Viability remained >85% for all control and device conditions (**Figure 4F**) and >95% at shorter collagenase digestion intervals for the device, with differences between conditions reaching statistical significance (ART ANOVA, F_6,14_ = 10.43, p < 0.0002).

We also evaluated static operation of the device for 60 min using collagenase (see **Supplementary Information**, **Supplementary Figure 3**). Although recovery differed across interval conditions for leukocytes, dendritic cells, and macrophages (ART ANOVA, p < 0.005 for each), total recovery after 60 min did not differ from static operation for any cell type (Dunnett’s test, p > 0.05). The same was found for viability for leukocytes, dendritic cells, and macrophages (ART ANOVA, p < 0.0002 for each dataset), with the 5 and 15 min intervals having higher viability than static operation (Dunnett’s test, p < 0.001 and p < 0.05, respectively), while the 60 min interval was similar (Dunnett’s test, p > 0.05). Thus, we conclude that continued recirculation through the device does not pose a significant concern for these cell subtypes when using collagenase, and static operation could be employed if a simpler scheme were desired.

### Combined dissociation protocol

**Figure 5:**
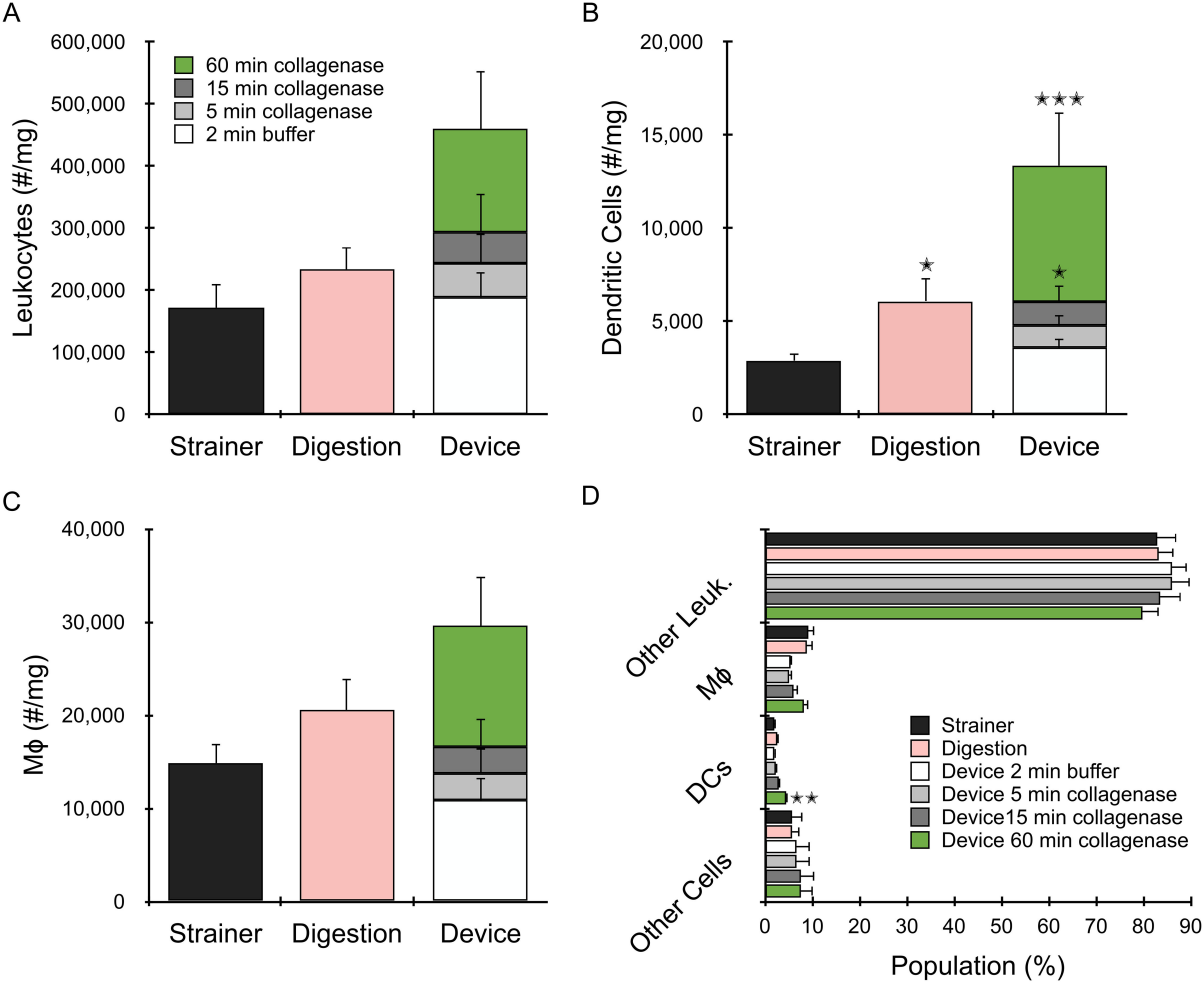
Combination protocol with non-enzymatic and enzymatic periods. Minced spleen was processed with buffer for 2 min followed by additional treatment with collagenase out to 60 min. Strainer and collagenase digestion controls were included for comparison. Cell recovery is presented for **(A)** total leukocytes, **(B)** dendritic cells, and macrophages. Device processing produced at least two-fold more cells for all subtypes. **(D)** Population percentages for each cell type. Dendritic cells and **(C)** macrophages were enriched at later times using the digestion device. Error bars represent standard error of the mean from n = 6 independent experiments. Mean tissue input: Strainer, 28.0 ± 3.9 mg; Digestion, 21.0 ± 3.2 mg; Device, 24.5 ± 1.8 mg. * indicates p < 0.05, ** indicates p < 0.01, and *** indicates p < 0.001 relative to collagenase control.

Next, we developed an operational scheme combining both non-enzymatic and enzymatic elements. We begin with a short 2 min interval using PBS+ buffer, which is intended to liberate large numbers of non-adherent or loosely adherent cells without exposure to enzyme. We then switch to collagenase to liberate cells that are more strongly adherent or located in deeper regions within minced spleen tissue. For this study, we included both non-enzymatic (strainer method) and enzymatic (60 min digestion with collagenase) controls for comparison. Cells were again analyzed by flow cytometry using the dendritic cell and macrophage panel. The strainer and collagenase controls produced ~170,000 and 230,000 leukocytes/mg tissue, respectively (**Figure 5A**). The increased yield with collagenase was statistically significant (p < 0.05), and consistent with reports that dendritic cells tend to be adherent, particularly when immature (37, 38), requiring digestion both for accessing more of the tissue and cell release. For the microfluidic device, the 2 min buffer interval released more leukocytes than the strainer control, confirming effective liberation of cells non-enzymatically. Notably, the 2 min buffer interval was only slightly less than the collagenase control, suggesting that most of the initial elution is due to mechanical forces. Collagenase was then used for intervals at 5, 15, 60 min total time. After the 5 min collagenase interval, total recovery was comparable to the collagenase control after 60 min, again demonstrating that hydrodynamic forces from the microfluidic device liberate cells more efficiently than standard methods. After the final 60 min collagenase interval, the total yield for the device was ~460,000 leukocytes/mg, which was >2-fold more than the collagenase control and >2.5-fold more than the strainer control. Results for dendritic cells again followed leukocyte trends, now exhibiting significant differences among processing conditions (ART ANOVA, F_5,30_ = 8.89, p < 0.0001). Total yield from the combined device protocol was ~13,000 cells/mg tissue (**Figure 5B**). This was >2- and >4.5-fold more than the collagenase and strainer controls, respectively, and differences were statistically significant (Šidák’s test, p < 0.0001). For macrophages, the total device yield was ~30,000 cells/mg tissue (**Figure 5C**), representing an ~2-fold increase relative to the strainer control; however, this difference did not reach significance in *post hoc* comparisons despite a significant overall effect for processing condition (ART ANOVA, F_5,30_ = 3.14, p = 0.0121). While the device yielded 1.5-fold more cells than the collagenase control, the difference was not significant. This result was not consistent with **Figure 4E**, and may in part be due to tissue pieces or cellular aggregates being eluted during the 2 min buffer interval, which will not break down as effectively without collagenase. Thus, additional dissociation of these aggregates via chemical digestion or enhanced mechanical dissociation may be required.

Population percentages for macrophages, dendritic cells, other CD45+ leukocytes, and other cells, as determined by flow cytometry, are shown in **Figure 5D**. RBCs, aggregates, and debris were excluded from analysis. Population percentages match previous reports in the literature (39, 40), suggesting that device processing is complete and does not bias towards any cell subtype. For both dendritic cells and macrophages, relative percentages generally increased with processing time. This suggests these cell subtypes were enriched, either because they were more effectively liberated at this time point and/or other cell subtypes were removed at earlier intervals. For the 60 min interval, significant differences emerged among comparisons (ART ANOVA, F_5,30_ = 3.70, p = 0.0100), with dendritic cells significantly enriched when compared to the strainer control (Šidák’s test, p = 0.0076), as well as being 2-fold higher than the 2 min interval using only buffer. This suggests that elution time could possibly serve as a means to crudely select for one cell subpopulation over others. For both strainer and collagenase digestion controls, viabilities were generally ~75-85% (ART ANOVA, p < 0.0001 for each dataset, see **Supplementary Information**, **Supplementary Figure 4**). Viability for device conditions tended to be higher at ~85-95%, again with modest decreases with processing time.

**Figure 6:**
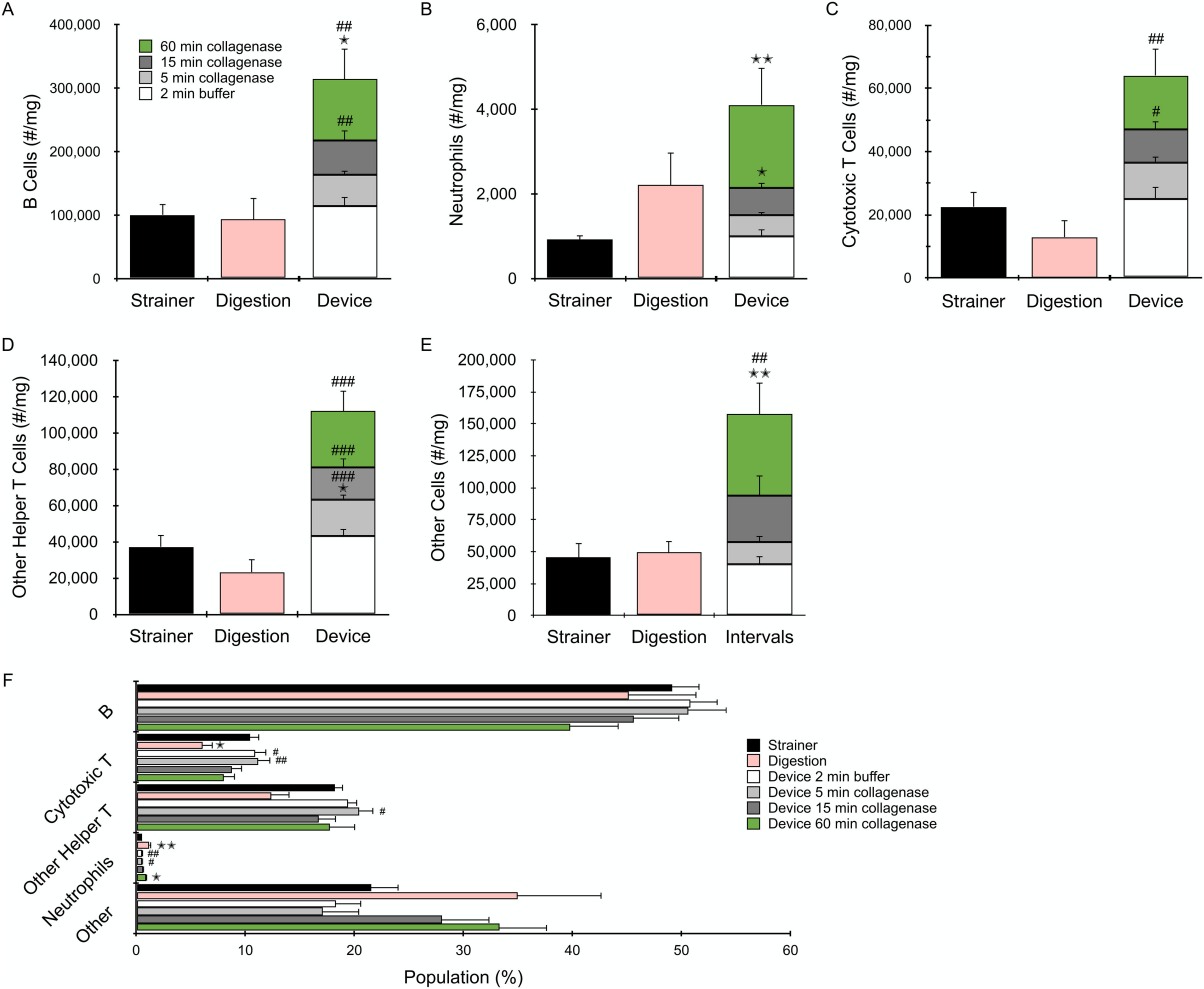
Evaluation of additional cell subtypes. Minced spleen tissue was processed as described in **Figure 4**, and total cell recovery is presented for **(A)** B cells, **(B)** neutrophils, **(C)** cytotoxic T cells, **(D)** other T cells, and **(E)** other cells. Again, the digestion device produced significantly more of each cell subtype, by 2 to 3-fold. **(F)** Population percentages, showing that all cell subtypes except neutrophils were enriched at later time points for the digestion device. Error bars represent standard error of the mean from n = 5 independent experiments. Mean tissue input: Strainer, 25.5 ± 6.0 mg; Digestion, 22.1 ± 3.7 mg; Device, 23.0 ± 3.7 mg. * indicates p < 0.05, ** indicates p < 0.01, and *** indicates p < 0.001 relative to strainer control. # indicates p < 0.05, ## indicates p < 0.01, and ### indicates p < 0.001 relative to collagenase control.

To gain further insight, we created a second flow cytometry panel that included T cells (general CD3+, cytotoxic CD8+), B cells (CD19+), and neutrophils (CD11b+ and Ly6G+), as defined in **Tables 3**, **4**. These surface markers were generally unaffected by collagenase digestion (see **Supplementary Information**, **Supplementary Figure 5**). We did note a small decrease in signal for CD19 and CD8a, but this did not affect proper identification of B and T cytotoxic T cells (see **Supplementary Information**, **Supplementary Figure 6**). For B cells, device recovery exhibited significant differences among comparisons (ART ANOVA, p = 0021), with total device recovery at ~310,000/mg, which was >3.4-fold more than both the strainer and collagenase controls (**Figure 6A**), and differences were statistically significant (Šidák’s test, both p < 0.05). Moreover, comparable number of B cells were released within the 2 min buffer interval as both controls, which is reasonable since B cells are non-adherent. However, the dramatic increase observed after using collagenase with the device suggests that a substantial number of B cells can be isolated from regions of spleen tissue that is otherwise inaccessible. For neutrophils, device recovery demonstrated significant differences among comparisons (ART ANOVA, p = 0002), with total device recovery resulting in ~4,000 cells/mg, which was >1.8- and 4.4-fold more than the collagenase digestion and the strainer controls, respectively (**Figure 6B**). The difference relative to the strainer control was statistically significant (Šidák’s test, p = 0.0002). Neutrophils primarily reside within the connective tissue of the red pulp, requiring digestion to effectively liberate these cells. Results for cytotoxic and non-cytotoxic T cells were similar to B cells, although the strainer controls outperformed collagenase controls in both cases. Total device recovery for cytotoxic T cells was ~64,000/mg, which were >2.8- and 5-fold increases compared to the strainer and collagenase digestion controls, respectively (**Figure 6C**). Significant differences were noted across processing conditions (ART ANOVA, p = 0.0033), and total device recovery was significantly higher than the collagenase digestion controls (Šidák’s test, p = 0.0044). Additionally, device recovery after the 15 minute interval was significantly higher than the collagenase controls (Šidák’s test, p =0.0156). We expect non-cytotoxic T cells to be predominantly helper T cells, and they very closely mirrored cytotoxic T cells, although with higher cell counts. The 2 min buffer interval yielded a greater number of cells than both the strainer and collagenase controls. Total device recovery was ~110,000 cells/mg, representing >3- and 4.8-fold more cells than the collagenase and strainer controls, respectively (**Figure 6D**). Recovery differed significantly across conditions (ART ANOVA, p < 0.0001), with the total device recovery statistically different than both strainer and collagenase digestion controls (Šidák’s test, p < 0.0001). For other cells that were not detected directly by our flow cytometry panel, differences between conditions were statistically significant (ART ANOVA, p = 0.001), with total device recovery (~160,000 cells/mg) ~3.2- and 3.5-fold more than the collagenase digestion and the strainer controls, respectively (**Figure 6E**). Total device recovery differences were statistically significant compared to both strainer and collagenase digestion controls (Šidák’s test, both p < 0.01).

**Table 3:** Secondary flow cytometry panel.

Marker	Fluorophore	Clone
CD3	BV421	17A2
Ly6G	BV510	1A8
CD8a	AF488	53-6.7
CD19	PE	6D5
TER119	AF647	TER-119
CD11b	APC/Cy7	M1/70
Viability	7-AAD	N/A

**Table 4:** Cell types identified using secondary flow cytometry panel.

Cell type	Surface markers
T cells	CD3+, CD19-, TER119-
Cytotoxic T cells	CD3+, CD8a+, CD19-, TER119-
Other T cells (helper T)	CD3+, CD8a-, CD19-, TER119-
B cells	CD19+, CD3-, TER119-
Neutrophils	CD11b+, Ly6G+, CD3-, CD19-, TER119-

Population percentages of B cells, cytotoxic T cells, other T cells, neutrophils, and other cells are shown in **Figure 6F**, and again aligned with published reports (39, 40). B cells, cytotoxic T cells, and other T cells were most enriched in the 2 and 5 min intervals, then generally decreased with processing time. As these cell types are generally non-adherent and primarily housed in the white pulp, they are more likely to be mechanically dissociated at early times. This is supported by the strainer control, which was more enriched with these cell types than the collagenase control. While B cells did not exhibit significant differences among conditions, cytotoxic T cells showed significant increases at both the 2 min and 5 min (combined) intervals compared to collagenase digestion (ART ANOVA, p = 0.0042; Šidák’s test, p < 0.05), whereas other T cells exhibited a significant increase only at the 2 min interval (ART ANOVA, p = 0.0416; Šidák’s test, p < 0.05). Neutrophils have the highest relative population percentages in the collagenase control and the 60 min device interval (ART ANOVA, p = 0.0009), which were both significantly higher than the strainer control (Šidák’s test p = 0.0030 for collagenase control, p = 0.0429 for 60 min device interval). This further suggests that collagenase digestion is needed to gain access to the connective tissue in the red pulp, where neutrophils primarily reside.

Viability for cell subtypes are presented in **Supplementary Information**, **Supplementary Figure 7**. All device conditions produced was >90% viable cells (ART ANOVA, p < 0.02 for all cell subtypes), while the strainer and collagenase controls were generally <90%, and in some cases <80%. Reduced viability for the strainer control was most pronounced for neutrophils (70%) and other T cells (80%). Collagenase controls had the lowest viability for cytotoxic T cells (65%), B cells (66%), and other T cells (77%). Taken together with the cell recovery data, these results suggest that the microfluidic device can isolate cell subtypes in both higher numbers and quality.

### Evaluation of B cell function

**Figure 7:**
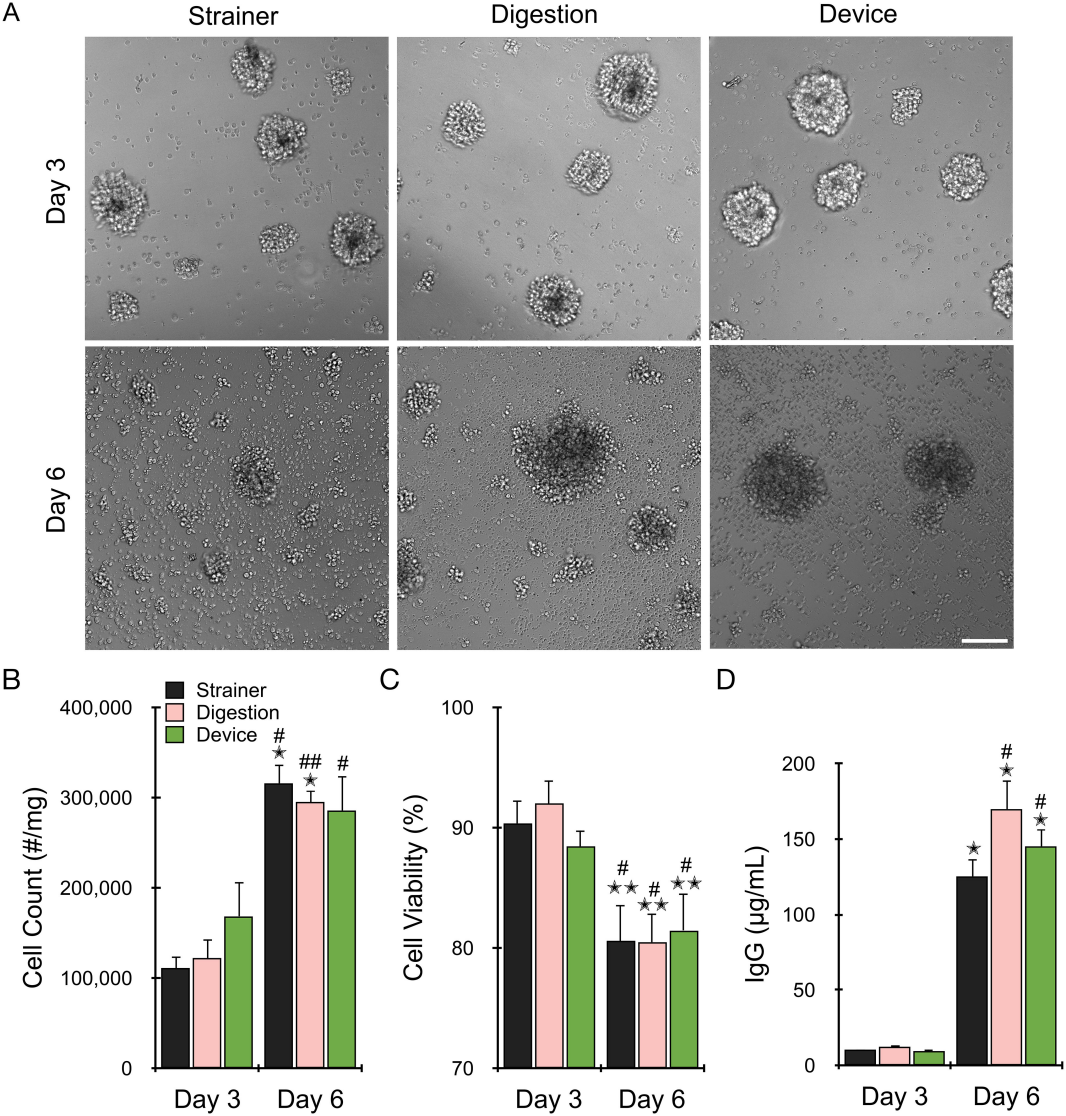
B cell growth and function. Minced spleen tissue was processed using the strainer control, digestion control, or microfluidic device using the final protocol (20 mL/min; 2 min buffer, collagenase out to 60 min). B cells were the sorted and cultured out to 6 days. **(A)** Phase micrographs of each condition after 3 and 6 days in culture, showing successful colony formation. **(B)** Cell count and **(C)** viability were assessed were comparable across conditions at the 3 and 6 day timepoints. **(D)** IgG secretion was quantified from conditioned media collected on days 3 and 6 using a mouse IgG ELISA. All conditions resulted in >12-fold increase in IgG secretion by day 6. Error bars represent standard error of the mean from n = 3 independent experiments. * indicates p < 0.05 and ** indicates p < 0.01 relative to strainer control. # indicates p < 0.05 and ## indicates p < 0.01 relative to collagenase control. Scale bar = 100 um.

Finally, B cells were assessed for growth in culture and IgG secretion to confirm longer-term and phenotype after processing with the microfluidic platform. B cells are both highly abundant and of critical importance for production of monoclonal antibodies in life sciences, biotechnology, and medicine. B cells were obtained using the microfluidic platform using the final combined protocol (buffer for 2 min; collagenase intervals at 5, 15, and 60 min), and all intervals were pooled. Both strainer and collagenase controls were tested for comparison. Upon collection, B cells were isolated by fluorescence-activated cell sorting (FACS), counted, seeded into 96-well plates, and cultured. Cell count, viability, and images were obtained on days 3 and 6 of culture. Colony formation was observed for all processing conditions (**Figure 7A**). Cell counts were higher on day 3 for the microfluidic platform compared to both strainer and collagenase digestion controls, although differences were not statistically significant (**Figure 7B**). By day 6, all groups had comparable cell counts but differences among groups were significant (ART ANOVA, p = 0.0017). All day 6 conditions demonstrated a significant increase in cell counts compared to the digestion control (Šidák’s test, p < 0.04 for all) while only the strainer and digestion controls were significant compared to the day 3 strainer (Šidák’s test, p < 0.02 for all). Viability was equivalent for all conditions on both days 3 and 6 at ~90% and 80%, respectively (ART ANOVA, p = 0.0008) (**Figure 7C**). The decrease in viability on day 6 was significant compared to both day 3 strainer (Šidák’s test, p < 0.008) and digestion (Šidák’s test, p < 0.02) controls. IgG concentration was assessed by ELISA using supernatant from 96-well plates collected on days 3 and 6. Results were converted to IgG concentration using a standard curve (see **Supplementary Information**, **Supplementary Figure 8**), and then normalized to the strainer control on day 3. Normalized results demonstrated comparable antibody production for all conditions (**Figure 7D**), with significant changes in IgG concentration after day 6 (ART ANOVA, p = 0.0015). Day 6 IgG concentration was significantly higher for all conditions compared to day 3 strainer (Šidák’s test, p < 0.05 for all) and both digestion and device conditions were significant compared to day 3 digestion (Šidák’s test, p < 0.05 for all). The collagenase control yielded slightly higher values on both days, but differences were not statistically significant. Taken together with the results from **Figure 6A**, microfluidic device processing yields greater B cell numbers without adversely affecting cell proliferation, viability, or antibody production.

## Materials and methods

### Digestion device

The microfluidic digestion device with luer opening was described previously. Briefly, PMMA and PET layers were laser machined using CO2 lasers to form fluidic channels, vias, and ports for luer connectors. Nylon mesh membranes (15 and 50 μm pore sizes; Amazon Small Parts) were laser machined to size. Devices were manufactured by Aline, Inc, where layers and components were aligned, adhesively bonded, and pressure-laminated to produce monolithic assemblies (31). Devices were prepared for operation by affixing 0.05” ID tubing (Saint-Gobain, Malvern, PA) to the device inlet and outlet hose barbs. 0.05” ID tubing was then connected to an Ismatec peristaltic pump (Cole-Parmer, Werheim, Germany) with 2.62 mm ID tubing (Saint-Gobern, Malvern, PA). Prior to experiments, devices and tubing were incubated with SuperBlock (PBS) blocking buffer (Thermo Fisher Scientific, Waltham, MA) at room temperature for 15 min to reduce non-specific binding of cells to channel walls, and then rinsed with PBS + 1% BSA (PBS+). Minced pieces of murine spleen tissue were loaded into the device tissue chamber through the luer inlet port. Devices and all tubing were then primed with the recirculation fluid, either PBS+ for non-enzymatic formats or 0.25% collagenase type I (Stemcell Technologies, Vancouver, BC) for enzymatic formats, and the luer port was closed off using a stopcock. For enzymatic formats, the entire experimental setup consisting of the device, tubing, and peristaltic pump were placed inside an incubator at 37 °C in order to maintain optimal enzymatic activity. The PBS+ or collagenase fluid was recirculated through the device and tubing using the peristaltic pump at a flow rate of 10 or 20 mL/min for the specified time. Flow rates above 20 mL/min were not evaluated, as the test range was selected based on prior work in other tissue types (31), and 20 mL/min represented the upper operating limit of the peristaltic pump. Intervals involved collecting the cell suspension from the device after the specified time, while replacing the volume with fresh buffer or collagenase solution. Additional processing was then performed for another interval period.

### Murine spleen specimen

Tissue preparation closely follows methods previously described for murine tissues such as kidney, liver, heart, and tumor (30, 31, 41). All mice were approximately 6–9 months of age. Both sexes were represented, although sex was not controlled as a biological variable. Unless otherwise indicated, all experiments were performed using n = 3 mice. Spleens were uniformly minced with a scalpel to ~1 mm^3^ pieces and weighed. Tissue mass per sample was recorded and reported in figure legends, as lower tissue inputs may introduce variability in normalized cell counts despite mass-based normalization. Device samples were processed as described above. For strainer controls, minced tissue was placed on a 100 μm cell strainer atop a 50 mL conical tube. The end of a 10 mL syringe plunger was then used to grind the tissue, and PBS+ was used to repeatedly flush cells through the strainer for collection. For collagenase digestion controls, minced tissue was placed within microcentrifuge tubes, digested at 37 °C using 0.25% type I collagenase solution in a shaking incubator under gentle agitation for 60 min, and mechanically disaggregated by repeated pipetting and vortexing. Finally, all cell suspensions were treated with 100 units of DNase I (Roche, Indianapolis, IN) for 10 min at 37 °C and washed by centrifugation into PBS+ (31).

### Flow cytometry

Cell suspensions were aliquoted, stained, filtered through 35 µm strainer tubes (Thermo Fischer Scientific, Catalog No. 08-771-23), and analyzed in accordance with the flow cytometry panels shown in **Tables 1**, **3**. For all studies, cell suspensions were first treated with 10 ug/mL TruStain FcX (anti-mouse CD16/32 clone 93) antibody (BioLegend, San Diego, CA) on ice for 10 min to block FC receptors and prevent nonspecific cell staining. For initial studies, cell suspensions were stained concurrently with 5 μg/mL anti-mouse CD45-BV510 (clone 30-F11, BioLegend, San Diego, CA) and 5 μg/mL TER119-AF647 (clone TER-119, BioLegend, San Diego, CA) monoclonal antibodies for 30 minutes at 4 °C in the dark. In later tests, cell suspensions were additionally stained with 10 ug/mL F4/80-PE (clone BM8), 2.5 ug/mL CD11c-FITC (clone N418), and 2.5 ug/mL MHC class II-APC/Cy7 (clone M5/114.15.2) antibody conjugates (all from BioLegend, San Diego, CA). For final evaluations with the expanded panel, a second aliquot of each test condition was stained concurrently with 2.5 ug/mL CD3-BV421 (clone 17A2), 5 ug/mL Ly6G-BV510 (clone 1A8), 2.5 ug/mL CD8a-AF488 (clone 53-6.7), 2.5 ug/mL CD19-PE (clone 6D5), 5 μg/mL TER119-AF647 (clone TER-119), 2.5 ug/mL CD11b-APY/Cy7 101226 (clone M1/70) antibody conjugates (all from BioLegend, San Diego, CA). For all panels, samples were washed using PBS+ by centrifugation (500 x g, 5 min), stained with 3.33 μg/mL viability dye 7-AAD (BD Biosciences, San Jose, CA) on ice for at least 10 minutes, and analyzed on a Novocyte 3000 Flow Cytometer (ACEA Biosciences, San Diego, CA). Flow cytometry data was compensated using single stained cell samples and/or compensation beads (Invitrogen, Waltham, MA). Gates encompassing positive and negative subpopulations within each compensation sample were inputted into FlowJo (FlowJo, Ashland, OR) to automatically calculate the compensation matrix. A sequential gating scheme was used to identify live and dead single immune cells from red blood cells, non-cellular debris, and cellular aggregates (**Supplementary Figures S9**, **S10**). Gating was established using strainer control samples and subsequently applied consistently across all experimental conditions. Signal positivity was determined using appropriate Fluorescence Minus One (FMO) controls. Control samples were left unstained.

### B cell culture and antibody production

B cells were isolated using the strainer control, collagenase digestion control, or microfluidic platform. Upon collection, RBCs were lysed using ACK lysis buffer and remaining cells were stained with TruStain FcX to block CD16/32 and reduce non-specific binding. Cells were then stained with monoclonal antibodies for CD3, CD19, CD45, and TER119 (**Table 3**) to sort for B cell isolation using a BD FACSAria III. B cells were identified by sequential gating to exclude debris and aggregates, and gate on live (TER119-) events. CD45^+^ cells were then selected, followed by gating for CD3^-^/CD19^+^ cells. After sorting, B cells were centrifuged, resuspended in ImmunoCult™ Mouse B Cell Expansion Kit (Stem Cell Technologies, Canada), and counted using an automated cell counter. Cells were then seeded into 96-well plates at 25,000 cells/well in triplicate per condition. During passaging, well components were transferred to a V bottom plate (Corning Costar) and centrifuged at 400 x g for 5 minutes. Supernatant was then collected in a separate V bottom plate, covered with a plate sticker (Axygen), and stored at -80C for later analysis. Cell density was adjusted to 25,000 cells for addition culture, and similar passaging was performed every third day. B cells were imaged at baseline (0 h) and every 3 days thereafter at 20x magnification using an Olympus IX83 microscope. Cell counts and viability were obtained every 3 days during passaging using an automated dual-fluorescence cell counter and AO/PI viability dye (Logos Biosystems Inc., Annandale, VA, USA). A mouse IgG ELISA kit (Abcam) was utilized to perform a sandwich ELISA on supernatant samples on days 3 and 6. A 4-parameter logistic regression was utilized to create a standard curve of mouse IgG standard included within the kit. Mouse IgG concentrations were calculated by interpolating absorbance values to replicate standard curves and correcting for sample dilution (1:150). Device and collagenase conditions were normalized to the mean day 3 strainer concentration.

### Statistics

Data are represented as the mean ± standard error. Error bars represent the standard error from at least three independent experiments. Datasets were analyzed using an aligned rank transform (ART) ANOVA using the ARTool package in RStudio (R version 4.4.1), followed by either a Dunnett’s multiple comparisons test when comparing to a single control or a Šídák’s multiple comparisons *post hoc* test for multiple control groups. Statistical significance was defined as p < 0.05.

## Conclusion

In this work, we have demonstrated that dissociation of murine spleen with our microfluidic digestion device can isolate the major cell subtypes more effectively than traditional methods. We demonstrated that higher flow rate and the interval operation enhanced leukocyte recovery without adversely affecting viability under enzymatic, non-enzymatic, and even combined operational formats. Under non-enzymatic conditions, which can be employed to preserve cell surface markers, the device matched or exceeded CD45+ leukocyte yield relative to the commonly used strainer control method. Device operation under enzymatic conditions greatly increased CD45+ leukocyte and adherent cell yields compared to non-enzymatic conditions. Using the combined protocol, with a non-enzymatic buffer washout followed by collagenase digestion, we showed that device processing resulted in higher yields and viabilities for dendritic cells, macrophages, B cells, neutrophils, and T cell subpopulations compared to controls. Functional validation in this study was limited to B cells, which exhibited similar proliferation, viability, and antibody production level to controls in culture. Future work will therefore extend functional validation to other key populations isolated by the device, including T cells, dendritic cells, and macrophages. Regarding throughput, the current device can be loaded with 20–30 mg of minced spleen tissue. Based on cell yields reported, this corresponds to ~10–20 million leukocytes per 60 min run. This could be expanded by running multiple devices in parallel. Future work will also focus on increasing chamber capacity to enable processing of larger tissue masses per device. In future work, we will explore additional device digestion protocols, including alternative proteolytic enzymes (i.e. collagenase D or type II, cocktails such as Liberase), higher flow rates, and more intervals to optimize cell recovery and function. Additionally, we will seek to evaluate cell suspensions isolated from spleen at higher resolution using methods such as single cell RNA sequencing, which will allow us to detect more cell subtypes (such as natural killer cells) and phenotypic states. Finally, we will explore clinically-relevant functional assays, including high-parameter immune phenotyping paired with cytokine multiplex profiling and T cell priming assays, to evaluate how our device can be utilized within a therapeutic workflow. This work will also advance biotechnology applications such as monoclonal antibody generation, cytotoxicity assays, vaccine testing, and adoptive cell transfer. Additionally, we will explore on-chip dissociation of other lymphoid tissues, such as the thymus and lymph nodes. Finally, we will integrate downstream technologies to enable on-chip immune cell sorting and single cell analysis.

## Data Availability

The raw data supporting the conclusions of this article will be made available by the authors, without undue reservation.
