# Supplementary material for: Microfluidic dissociation of spleen to recover immune cells for biotechnology and single cell analysis
Source: Front Immunol. 2026 Jul 29;17:1819465. doi: 10.3389/fimmu.2026.1819465 (PMC13461508; doi:10.3389/fimmu.2026.1819465)
Supplement: Supplementary file 1 [file DataSheet1.pdf]

Supplementary Information

Microfluidic dissociation of spleen to recover immune cells for biotechnology and single cell analysis

Jeremy A. Lombardo,[a,†] David Zalazar,[a,†] Marzieh Aliaghaei,[b] and Jered B. Haun*[abcfg]

[a.] Department of Biomedical Engineering, University of California Irvine, Irvine, CA 92697, USA.

[b.] Department of Chemical and Biomolecular Engineering, University of California Irvine, Irvine, CA 92697, USA.

[c.] Department of Materials Science and Engineering, University of California Irvine, Irvine, CA 92697, USA.

[d.] Chao Family Comprehensive Cancer Center, University of California Irvine, Irvine, CA 92697, USA.

[e.] Center for Advanced Design and Manufacturing of Integrated Microfluidics, University of California, Irvine, Irvine, CA, 92697, USA.

†These authors contributed equally to the work

*Jered B. Haun, PhD

Associate Professor

Department of Biomedical Engineering

University of California Irvine

3107 Natural Sciences II, Irvine, CA, 92697

Phone: 949-824-1243

E-mail: jered.haun@uci.edu

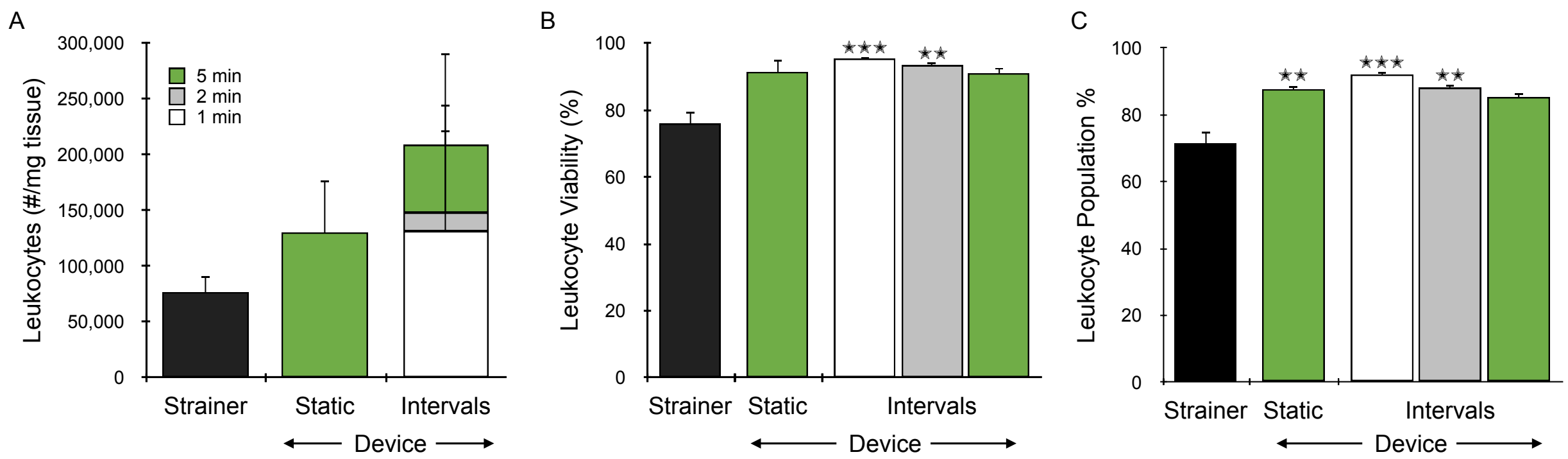

Figure S1: Static versus interval operation modes under non-enzymatic conditions. Minced spleen was processed using the strainer control, as well as the digestion device with buffer under static and interval modes. (A) Total leukocyte recovery was >2-fold higher for both device modes relative to the control. (B) Leukocyte viability was higher for all device conditions regardless of operational mode or interval timing. (C) Leukocyte population percentage was higher for 1-min and 2-min intervals compared to static and all device conditions compared to strainer. Error bars represent standard error of the mean from n = 3 independent experiments. Mean tissue input: Strainer, 22.6 ± 2.9 mg; Static, 16.4 ± 1.2 mg; Intervals, 19.5 ± 1.1 mg. ** indicates $p < 0.01$ and *** indicates $p < 0.001$ relative to strainer control.

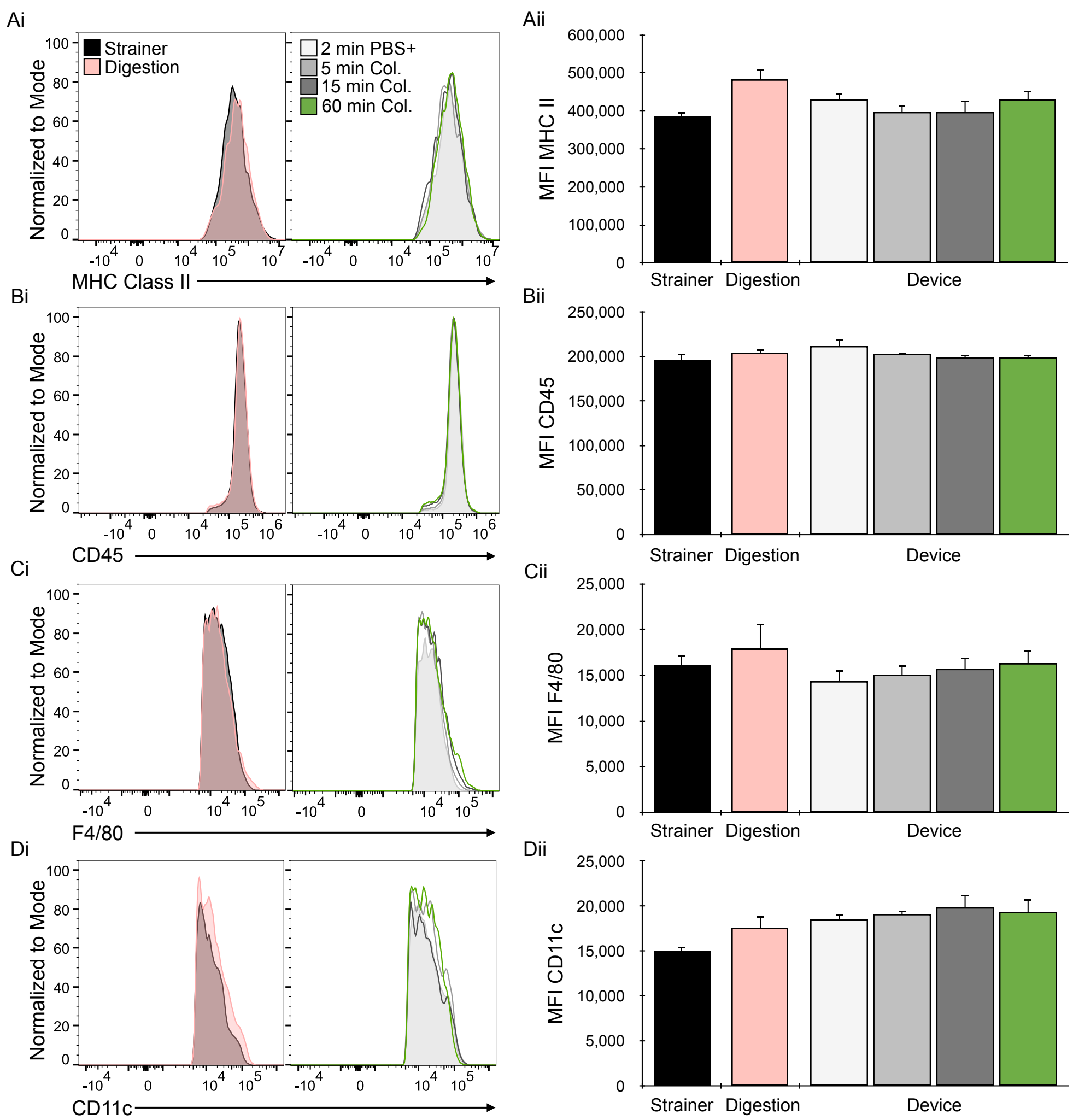

Figure S2: Surface marker expression is preserved with digestion and device treatment. Fluorescence intensity of (A) MHC Class II, (B) CD45, (C) F4/80 , and (D) CD11c was determined for strainer and collagenase digestion controls, as well as different device intervals (with and without collagenase. Results are presented as (i) histograms from one representative experiment and (ii) bar graphs of geometric mean fluorescence intensity across n=3 biological replicates for each condition. There was no change in signal intensity or distribution for any marker. MHC class II was assessed on live dendritic cells (CD45+, CD11c+hi, MHC II+, TER119−). CD45 was assessed on live singlets. F4/80 was assessed on live CD45+, TER119− cells. CD11c was assessed on live CD45+, MHC Class II+, TER119− cells.

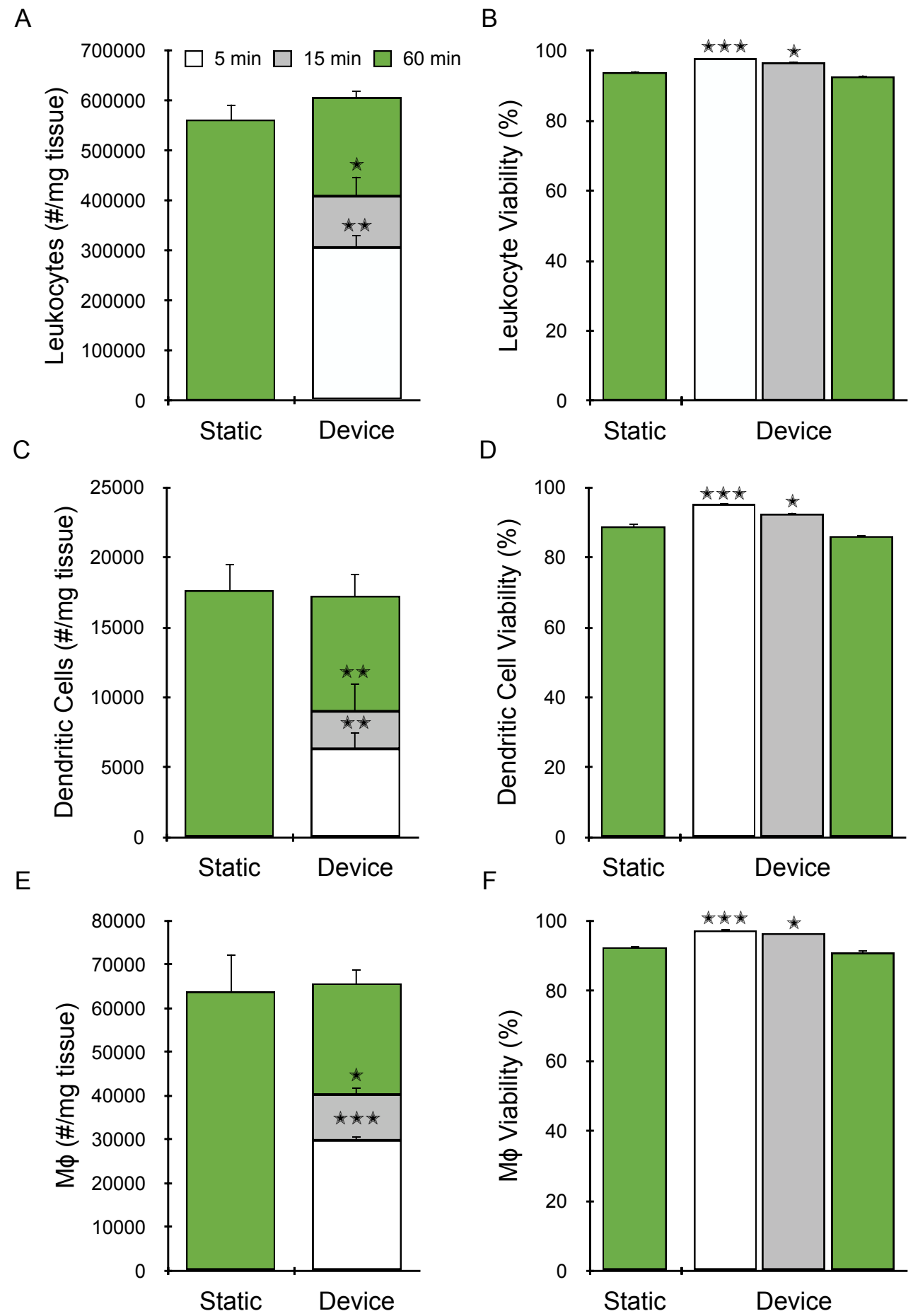

Figure S3: Static and interval operation modes under enzymatic conditions. Minced spleen was processed using the digestion device with collagenase under static and interval modes. Cell recovery and viability was similar for (A,B) total leukocytes, (C,D) dendritic cells, and (E,F) macrophages under all conditions. Error bars represent standard error of the mean from n = 3 independent experiments. Mean tissue input: Static, 23.6 ± 1.0 mg; Device, 22.4 ± 1.3 mg. * indicates $p < 0.05$, ** indicates $p < 0.01$, and *** indicates $p < 0.001$ relative to static device control.

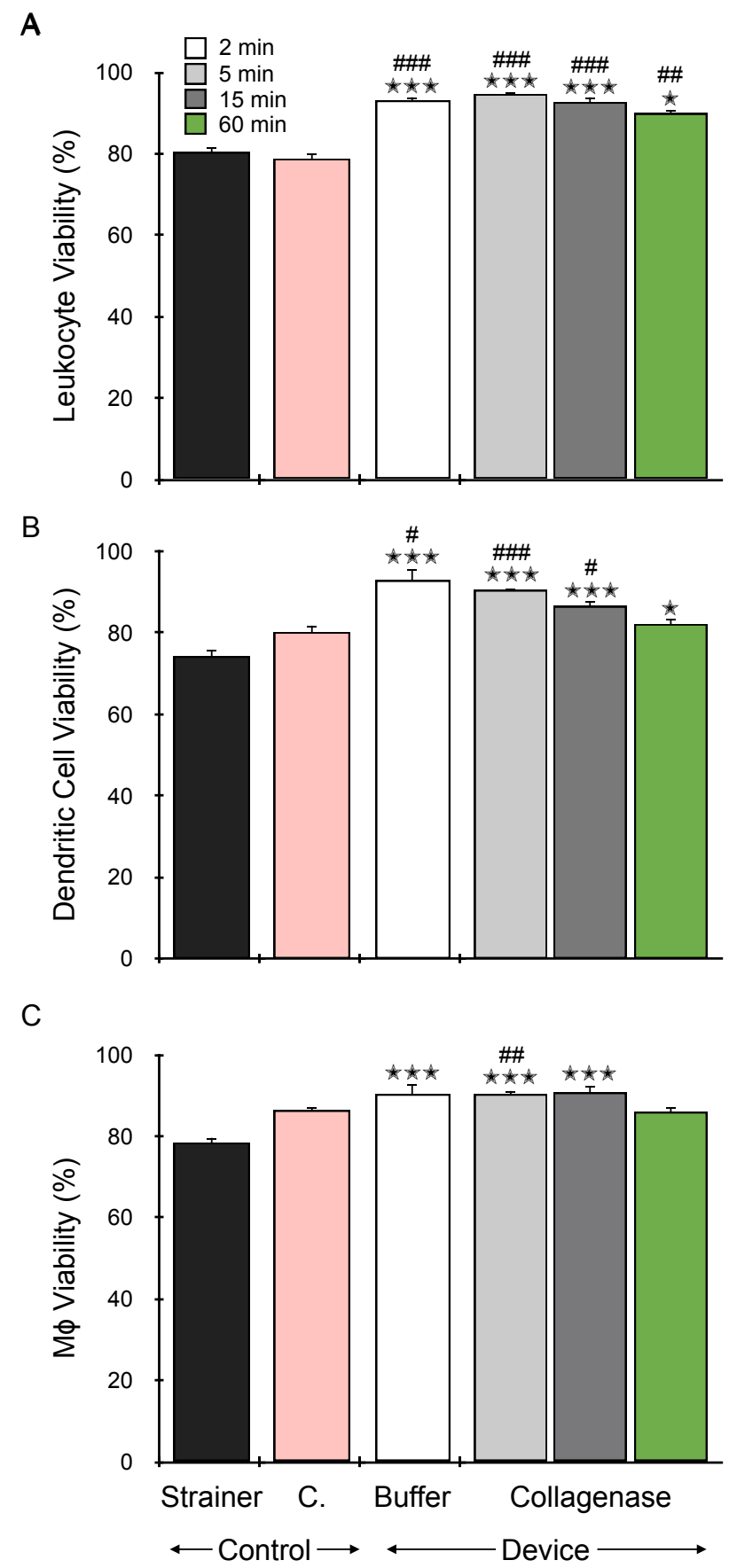

Figure S4: Cell viability for final combined protocol with first cell panel. Viability for (A) leukocytes, (B) dendritic cells, and (C) macrophages/monocytes were ~70-85% in controls and ~85-95% in device conditions. Error bars represent standard error of the mean from n = 6 independent experiments. * indicates $p < 0.05$ and *** indicates $p < 0.001$ relative to strainer control. # indicates $p < 0.05$, ## indicates $p < 0.01$, and ### indicates $p < 0.001$ relative to collagenase control.

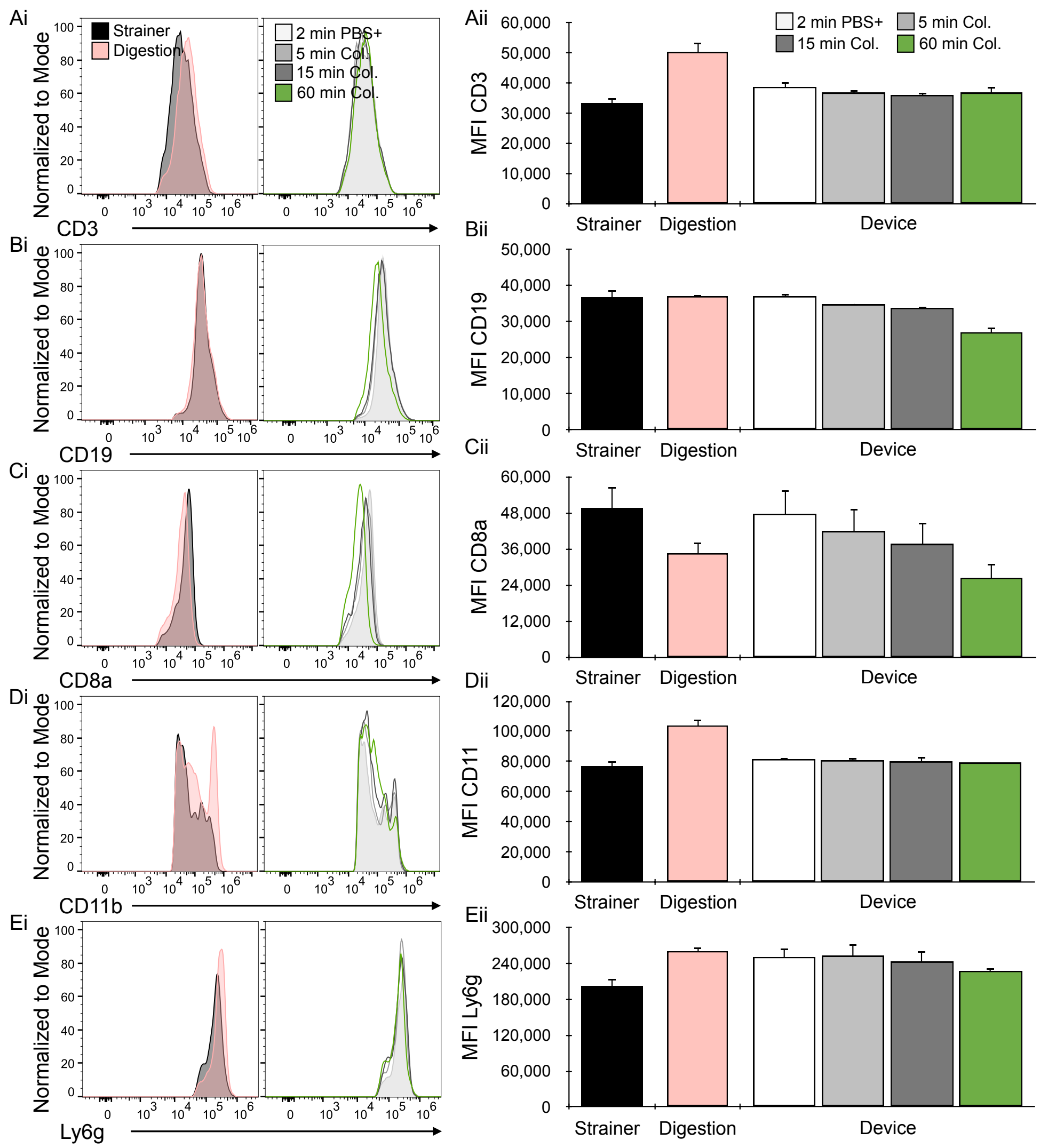
Ai
Strainer
Digestion
2 min PBS+
5 min Col.
15 min Col.
60 min Col.
Normalized to Mode
CD3
Aii
MFI CD3
Strainer
Digestion
Device
Bi
CD19
Bii
MFI CD19
Ci
CD8a
Cii
MFI CD8a
Di
CD11b
Dii
MFI CD11
Ei
Ly6g
Eii
MFI Ly6g

Figure S5: Secondary panel surface marker integrity is maintained across digestion and device conditions. Fluorescence intensity of (A) CD3, (B) CD19, (C) CD8a, (D) CD11b, and (E) Ly6G was determined for strainer and collagenase digestion controls, as well as different device intervals (with and without collagenase. Results are presented as (i) histograms from one representative experiment and (ii) bar graphs of geometric mean fluorescence intensity across n=3 biological replicates for each condition. There was no change in signal intensity or distribution for for CD3, CD11b, and Ly6G, but a small decrease in CD19 and CD8a were observed. All populations were gated within TER119−, live singlets. CD3 was assessed on CD19− cells. CD19 was assessed on CD3− cells. CD8a was assessed on cells gated as CD3+, CD19−. CD11b was assessed on cells gated as CD3−, CD19−. Ly6G was assessed on cells gated as CD11b+, CD3−, CD19−.

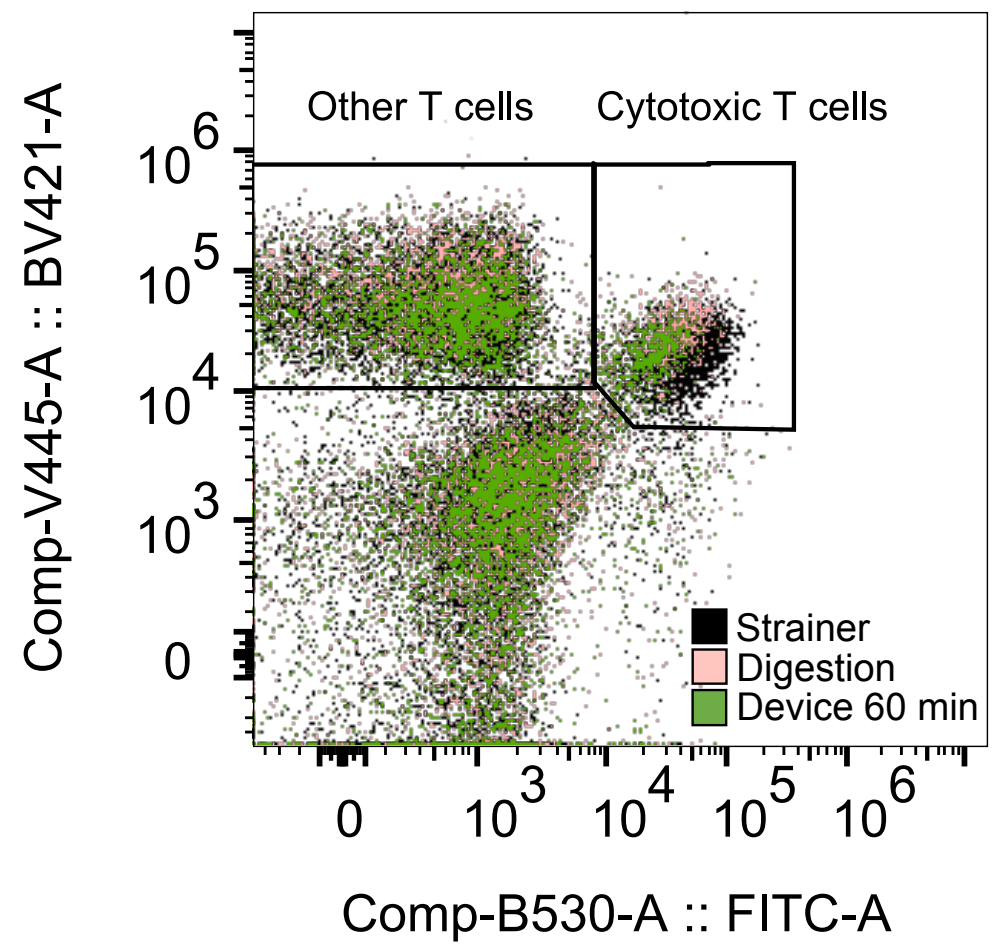

Condition	Other T cell Frequency	Cytotoxic T cell Frequency
Strainer PBS+	39.2%	16.1%
Control Collagenase	37.8%	8.76%
Device 60 min Collagenase	37.2%	7.44%

Figure S6: CD8a+ cytotoxic T cells remain within detection gate following collagenase digestion. Representative dot plot showing CD8a (FITC) vs CD3 (BV421) expression gated within CD19−, TER119−, live singlets. Gates identify other T cells (CD3+, CD8a−) and cytotoxic T cells (CD3+, CD8a+). Three conditions are overlaid: strainer control (black), collagenase digestion control (pink), and device 60 min interval with collagenase (green). Table shows frequency of parent values for a single representative replicate. While cytotoxic T cell frequency was reduced with collagenase exposure, the population remained clearly distinguishable from other T cells across all conditions.

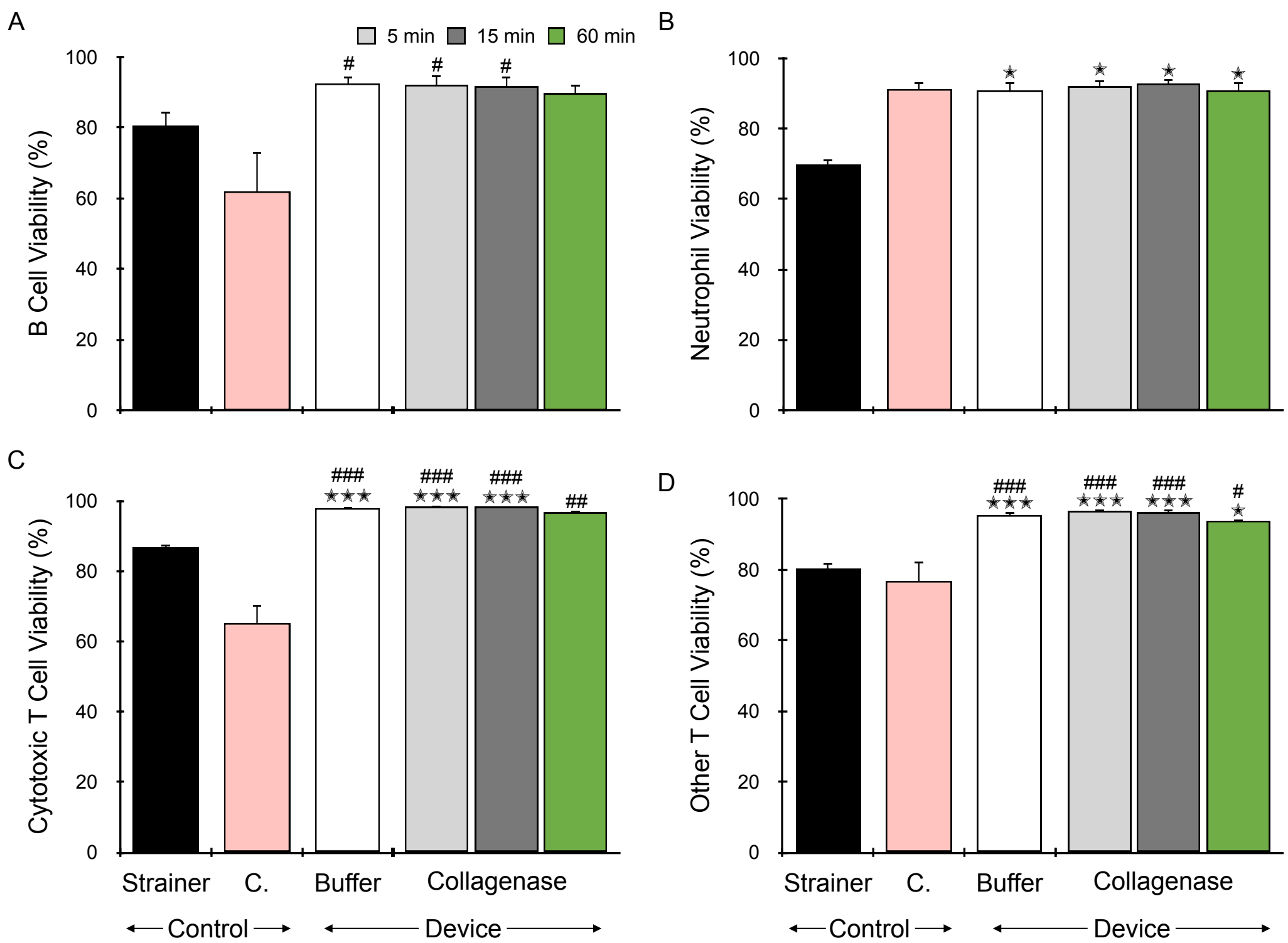

Figure S7: Cell viability for final combined protocol with second cell panel. Viability for (A) B cells, (B) granulocytes, (C) cytotoxic T cells, and (D) other T cells was >90% for all device intervals which matched or exceeded viability of the strainer and collagenase controls. Error bars represent standard error of the mean from n = 6 independent experiments. * indicates p < 0.05 and *** indicates p < 0.001 relative to strainer control. # indicates p < 0.05, ## indicates p < 0.01, and ### indicates p < 0.001 relative to collagenase control.

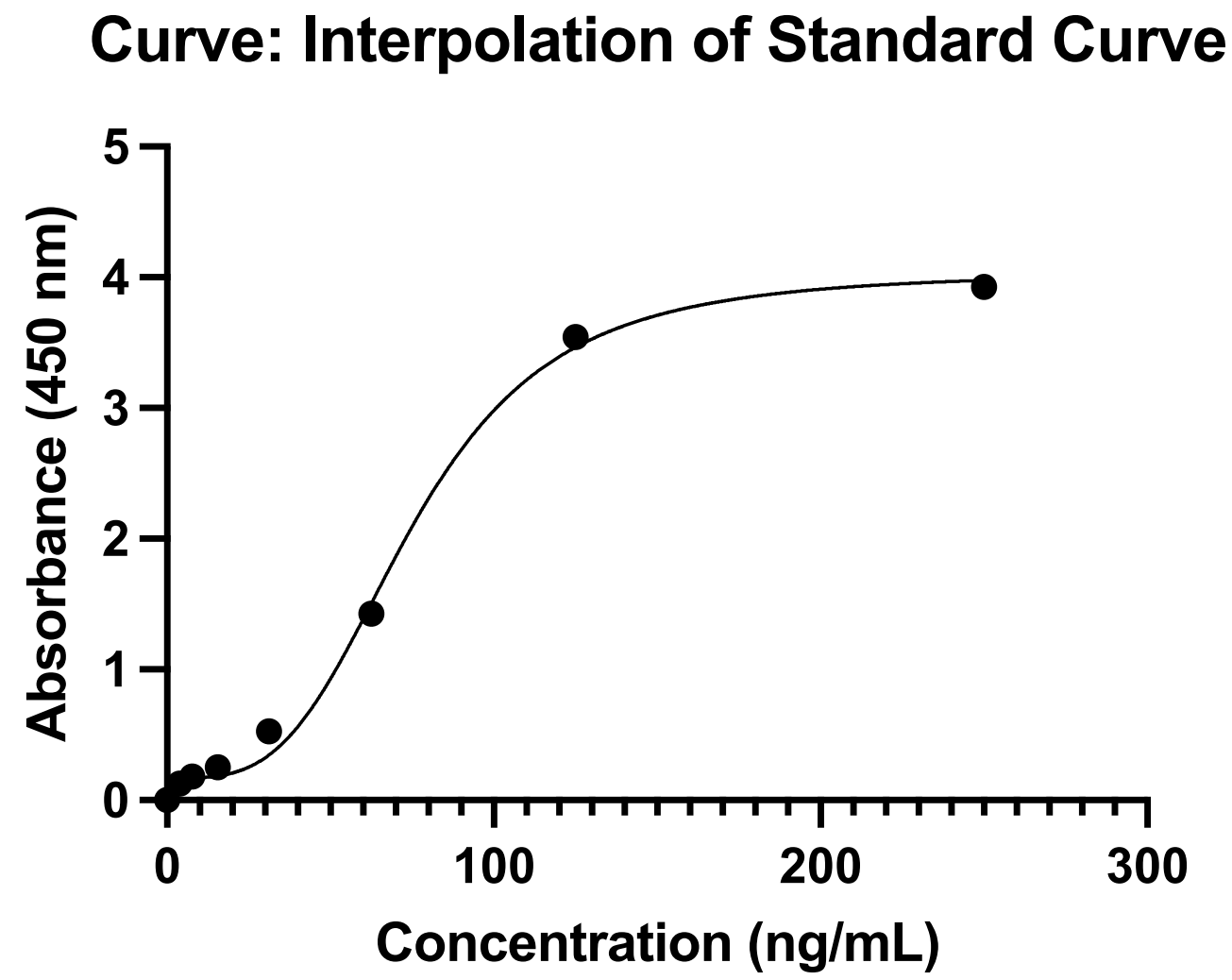

Figure S8: Standard curve for IgG ELISA. Absorbance at 450 nm plotted as a function of IgG concentration for a representative assay. A nonlinear regression fit was used to interpolate sample concentrations within the linear range of the curve.

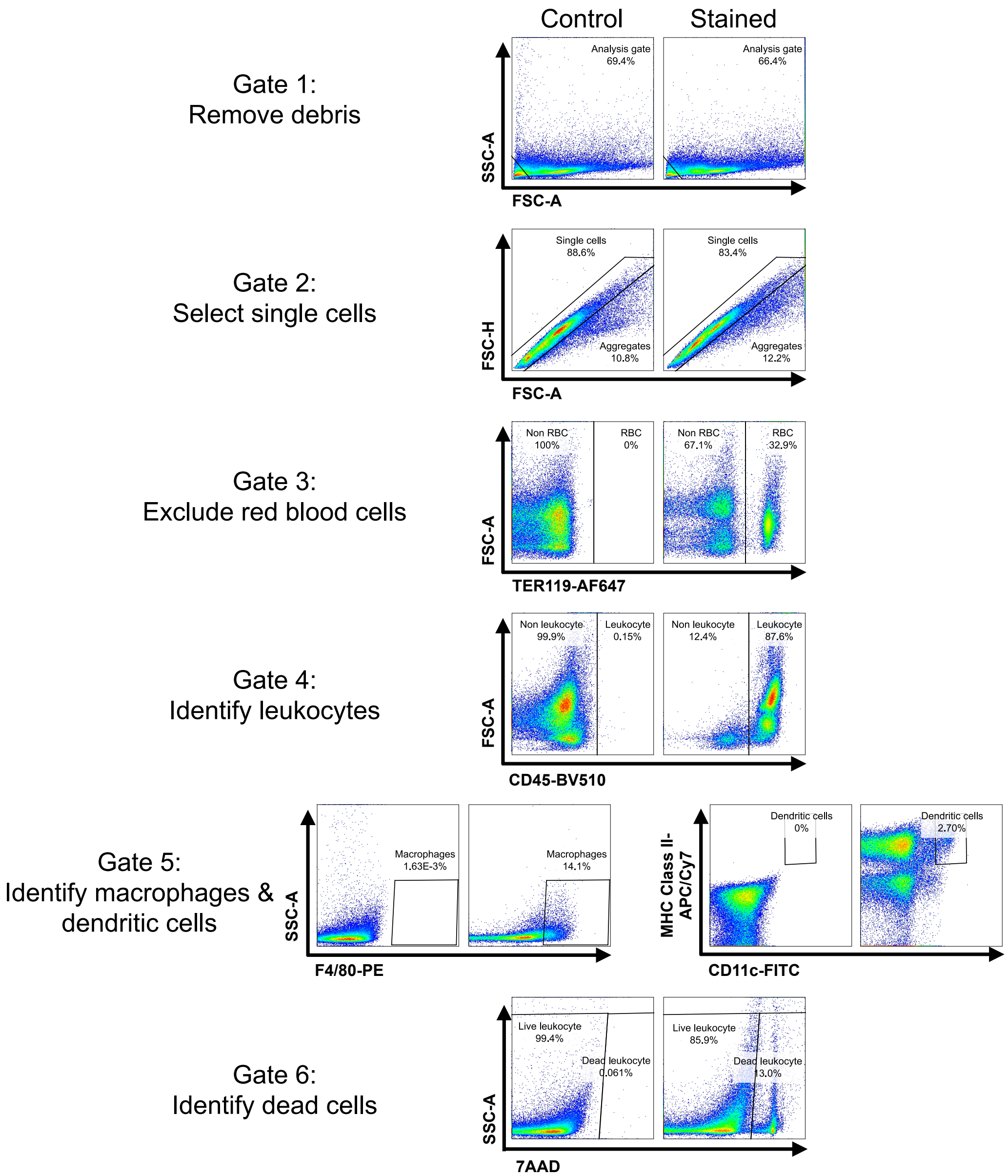
Control
Stained
Gate 1:
Remove debris
Analysis gate
69.4%
Analysis gate
66.4%
SSC-A
FSC-A
Gate 2:
Select single cells
Single cells
88.6%
Single cells
83.4%
Aggregates
10.8%
Aggregates
12.2%
FSC-H
FSC-A
Gate 3:
Exclude red blood cells
Non RBC
100%
RBC
0%
Non RBC
67.1%
RBC
32.9%
FSC-A
TER119-AF647
Gate 4:
Identify leukocytes
Non leukocyte
99.9%
Leukocyte
0.15%
Non leukocyte
12.4%
Leukocyte
87.6%
FSC-A
CD45-BV510
Gate 5:
Identify macrophages &
dendritic cells
Macrophages
1.63E-3%
Macrophages
14.1%
SSC-A
F4/80-PE
Dendritic cells
0%
Dendritic cells
2.70%
MHC Class II-
APC/Cy7
CD11c-FITC
Gate 6:
Identify dead cells
Live leukocyte
99.4%
Dead leukocyte
0.061%
Live leukocyte
85.9%
Dead leukocyte
13.0%
SSC-A
7AAD

Figure S9: Flow cytometry gating scheme for first cell subtype panel (Table 2). Cell suspensions obtained from dissociated spleen samples were stained with the fluorescent probes listed in Tables 1 and analyzed using flow cytometry. Acquired data was compensated and assessed using a sequential gating scheme. Gate 1 was based on FSC-A vs. SSC-A, and used to exclude debris near the origin. Gate 2 was was used to select single cells based on FSC-A vs. FSC-H. Gate 3 identified and excluded red blood cells based TER119-AF647 positive signal. Gate 4 identified leukocytes based on CD45-BV510 positive signal. Gate 5 was applied to the TER119-,CD45+ cell subset to identify macrophages based on positive F4/80-PE signal, and to identify dendritic cells based on positive MHC class II-APC/Cy7 and CD11c-FITC signals. Finally, gate 6 was used to identify live cells in leukocyte, macrophage, and dendritic cell subsets based on negative 7-AAD signal. Appropriate isotype controls were initially used to assess nonspecific background staining, and appropriate fluorescence minus one (FMO) controls were used to determine positivity and set gates. Control samples were left unstained.

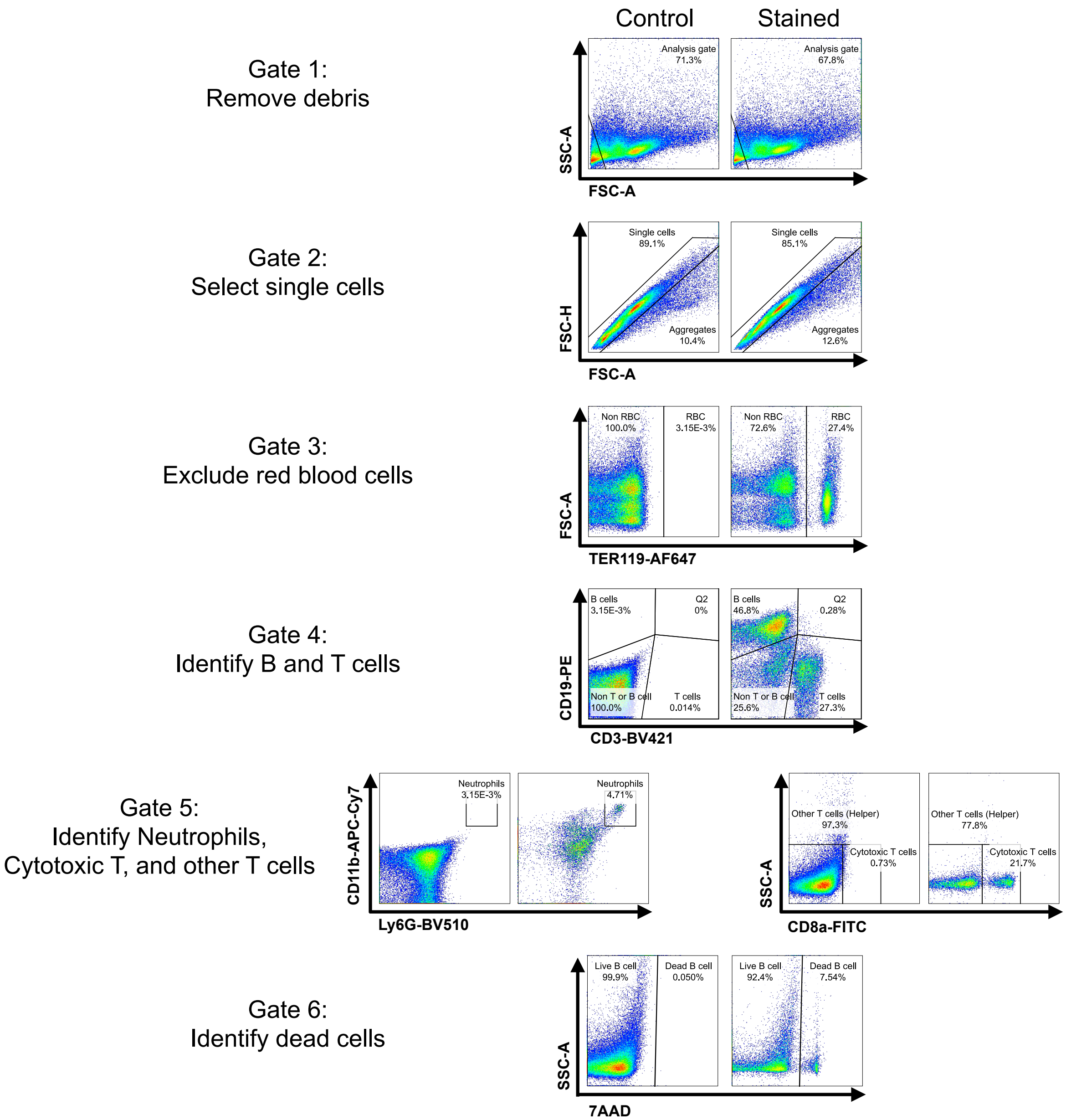

Control
Stained
Gate 1:
Remove debris
Analysis gate 71.3%
Analysis gate 67.8%
SSC-A
FSC-A
Gate 2:
Select single cells
Single cells 89.1%
Aggregates 10.4%
Single cells 85.1%
Aggregates 12.6%
FSC-H
FSC-A
Gate 3:
Exclude red blood cells
Non RBC 100.0%
RBC 3.15E-3%
Non RBC 72.6%
RBC 27.4%
FSC-A
TER119-AF647
Gate 4:
Identify B and T cells
B cells 3.15E-3%
Q2 0%
Non T or B cell 100.0%
T cells 0.014%
B cells 46.8%
Q2 0.28%
Non T or B cell 25.6%
T cells 27.3%
CD19-PE
CD3-BV421
Gate 5:
Identify Neutrophils,
Cytotoxic T, and other T cells
Neutrophils 3.15E-3%
Neutrophils 4.71%
CD11b-APC-Cy7
Ly6G-BV510
Other T cells (Helper) 97.3%
Cytotoxic T cells 0.73%
Other T cells (Helper) 77.8%
Cytotoxic T cells 21.7%
SSC-A
CD8a-FITC
Gate 6:
Identify dead cells
Live B cell 99.9%
Dead B cell 0.050%
Live B cell 92.4%
Dead B cell 7.54%
SSC-A
7AAD

Figure S10: Flow cytometry gating scheme for first cell subtype panel (Table 4). Cell suspensions obtained from dissociated spleen samples were stained with the fluorescent probes listed in Table 3 and analyzed using flow cytometry. Acquired data was compensated and assessed using a sequential gating scheme. Gate 1 was based on FSC-A vs. SSC-A, and was used to exclude debris near the origin. Gate 2 was then used to select single cells based on FSC-A vs. FSC-H. Gate 3 identified and excluded red blood cells based TER119-AF647 positive signal. Gate 4 was applied to the TER119- cell subset to identify B cells based on positive CD19-PE signal and negative CD3-BV421, and to identify T cells based on positive CD3-BV421 signal and negative CD19-PE signal. Gate 5 was applied to the CD3-,CD19- cell subset to identify granulocytes based on positive Ly6G-BV510 and CD11b-APC/Cy7 signals, and was applied to the CD3+, CD19- cell subset to identify cytotoxic T cells based on positive CD8a-AF488 signal. Finally, gate 6 was used to identify live cells in B cells, granulocytes, and T cell subsets based on negative 7-AAD signal. Appropriate isotype controls were initially used to assess nonspecific background staining, and appropriate FMO controls were used to determine positivity and set gates. Control samples were left unstained.
